# Generative AI in higher education: a meta-analysis of intellectual and social–emotional outcomes

**DOI:** 10.3389/fpsyg.2026.1848745

**Published:** 2026-06-08

**Authors:** Chunai Liu, Lidong Xie, Gang Xu

**Affiliations:** 1 School of Education, Chuxiong Normal University, Chuxiong, China; 2 School of Physical Education, Sichuan University of Science & Engineering, Zigong, China

**Keywords:** generative AI, higher education, intellectual outcomes, meta-analysis, social emotional outcomes

## Abstract

**Background:**

Generative AI has rapidly entered higher education, yet evidence on its effects across learning domains remains fragmented. This study synthesized experimental evidence on whether generative AI improves intellectual outcomes and social emotional outcomes among higher education students.

**Methods:**

A systematic review and meta-analysis was conducted on 33 experimental and quasi-experimental studies involving 3,394 participants. The included studies were published between 2021 and 2024, and the literature search encompassed the period from database inception to January 26, 2026. Random-effects models in Stata 18 were used to estimate pooled effects for intellectual outcomes and social emotional outcomes, and publication bias, sensitivity analyses, and moderator analyses were also performed.

**Results:**

Generative AI demonstrated a significant positive effect on intellectual outcomes (Hedges’ *g* = 1.096, 95% CI 0.087 to 2.104, *p* = 0.033) and a smaller but significant positive effect on social emotional outcomes (Hedges’ *g* = 0.301, 95% CI 0.048 to 0.553, *p* = 0.020). The difference between the two outcome domains was not statistically significant (coefficient = -0.739, *p* = 0.252). Publication bias was detected for intellectual outcomes but not for social emotional outcomes. Sensitivity analyses indicated that social emotional findings were relatively stable, whereas intellectual outcomes were more sensitive to individual studies. Moderator analyses showed that functional type, intervention duration, and knowledge domain did not significantly explain between-study heterogeneity at the omnibus level.

**Conclusion:**

Generative AI appears to be a promising educational resource in higher education, particularly for intellectual outcomes, although the current evidence base remains heterogeneous and methodologically uneven.

**Systematic review registration:**

PROSPERO CRD420261361622.

## 1 Introduction

Artificial intelligence has progressively emerged as a significant technological force in higher education; however, the rise of generative artificial intelligence (GAI) has reshaped this landscape in ways that differ markedly from earlier forms of educational artificial intelligence (Zawacki-Richter et al., 2019). In the initial stages of higher education applications, artificial intelligence was primarily associated with intelligent tutoring systems, learning analytics, predictive models, and automated decision support. More recently, systems built on large language models have demonstrated the capacity to generate natural language explanations, interactive feedback, content summaries, learning prompts, and original text in real time (Ouyang and Jiao, 2021; Kasneci et al., 2023). This transformation has broadened the pedagogical role of artificial intelligence from comparatively structured assistance to more open-ended, dialogic, and adaptive forms of support, thereby raising important new questions regarding how such tools shape student learning in higher education (Tlili et al., 2023).

In the context of higher education, GAI is considered to have many potential teaching values. Studies have pointed out that tools such as ChatGPT can provide instant interpretation, promote opinion generation, support text revision, deliver personalized feedback, and support more flexible learning processes outside the classroom (Lo, 2023; Grassini, 2023). At the same time, the same literature has raised substantial concerns regarding misinformation, hallucinated content, overreliance, reduced independent thinking, and academic integrity risks (Cotton et al., 2024). GAI has therefore quickly emerged as a highly visible educational technology in higher education, one that combines substantial pedagogical opportunities with considerable uncertainty regarding standards, reliability, and responsible use (Bin-Nashwan et al., 2023). The current core question is no longer whether such tools will enter higher education, but under what conditions their use can really promote rather than weaken meaningful learning.

Against this backdrop, evidence syntheses in this area have expanded rapidly. Recent systematic reviews and meta-analyses have moved beyond descriptive mapping and begun to quantify the educational effects of GAI and ChatGPT more directly. For example, Ma and Zhong reported a significant positive overall effect of GAI on learning outcomes, Xia et al. synthesized evidence on university students’ motivation and engagement, and Mo et al. further examined ChatGPT-related learning outcomes specifically among undergraduate students (Ma and Zhong, 2025; Qi et al., 2025; Mo et al., 2026). More recent 2026 meta-analyses by Doo and Wu et al. have continued this trend by reporting positive effects of GenAI or ChatGPT on educational outcomes, learning achievement, and broader student learning outcomes (Doo and Park, 2026; Wu et al., 2026). Collectively, these studies indicate that the quantitative evidence base is expanding rapidly, but they also suggest that the field remains methodologically heterogeneous and conceptually uneven.

Although this line of evidence is highly informative, the current meta-analytic literature remains heterogeneous in scope. Some syntheses examine broad artificial intelligence applications rather than GAI specifically (Torres and Kahveci, 2025), some combine learners across multiple educational levels rather than isolating higher education (Dong, 2026), and others pool conceptually diverse indicators into broad composite categories such as overall learning outcomes or combined cognitive and non-cognitive outcomes (Ma and Zhong, 2025). Even newer achievement-oriented syntheses often focus on ChatGPT alone or do not center higher education as a distinct population (Doo and Park, 2026). These scope decisions make it difficult to determine whether the reported benefits are specific to GAI, specific to university learners, or specific to particular outcome domains. This lack of analytical focus creates both conceptual and practical problems. Conceptually, it blurs the boundaries between different forms of artificial intelligence, different developmental stages of learners, and different categories of educational outcomes, thereby weakening interpretability and limiting theory building. Practically, it reduces the usefulness of the evidence for higher education instructors and institutions that need more precise guidance on whether GAI primarily supports performance-related intellectual outcomes (IOs), social–emotional outcomes (SOs), or both. The present meta-analysis moves away from these broader approaches by focusing specifically on GAI, restricting the population to higher education, and separating intellectual and social–emotional outcomes rather than collapsing them into a single composite indicator. This design allows for a more targeted evaluation of the educational role of GAI and provides clearer evidence for both research interpretation and pedagogical decision-making.

### 1.1 Conceptualizing IO and SO

In the present study, the distinction between IO and SO is central to the review framework. IOs refer to performance-oriented cognitive and academic consequences of learning, whereas social–emotional outcomes refer to affective, motivational, and self-perceptual dimensions of student learning. To clarify the conceptual distinction between these two domains and to summarize how they have typically been operationalized in prior research, Supplementary Table 1 presents the key outcome categories together with their representative constructs and common measurement indicators.

As shown in Supplementary Table 1, IO in prior GAI research have commonly been measured through indicators such as academic achievement, knowledge acquisition, writing performance, feedback quality, problem-solving, and critical thinking, often using test scores, rubric-based assessments, and task performance measures, whereas SO have typically been assessed through constructs such as motivation, engagement, attitudes, self-efficacy, enjoyment, anxiety, and willingness to communicate, usually through self-report questionnaires or validated affective scales. Because some educational constructs occupy the boundary between cognition and affect, their classification is not self-evident across studies. For this reason, the present review classifies outcomes according to their dominant theoretical function rather than their possible downstream consequences.

### 1.2 Potential moderators of GAI effects in higher education

The effects of GAI in higher education are also likely to vary according to specific study and intervention characteristics. For this reason, the present review considers several moderators that are theoretically relevant to how GAI is integrated into teaching and learning.

First, the functional type of GAI may shape its educational influence because different uses of GAI involve different forms of cognitive support and learner interaction. GAI can serve as a source of personalized learning support, an intelligent tutoring partner, a feedback provider, a writing assistant, or a task-oriented problem-solving tool. These functional differences may affect the degree of scaffolding, immediacy, personalization, and cognitive offloading available to students, which in turn may influence the magnitude and direction of learning outcomes.

Second, intervention duration may moderate the effects of GAI because the educational value of such tools may develop over time rather than appear immediately. Short-term interventions may capture novelty effects, initial engagement, or immediate task support, whereas longer interventions may better reflect sustained use, strategy adaptation, prompt literacy development, and the gradual integration of GAI into students’ self-regulated learning processes. Duration is therefore relevant not only methodologically but also pedagogically.

Third, the knowledge domain of the instructional context may influence the effects of GAI because disciplinary tasks differ in epistemic structure, communication demands, and criteria for successful performance. For example, GAI may function differently in language learning, programming, teacher education, design, law, or other disciplinary contexts because the nature of the task, the acceptable role of assistance, and the balance between creativity and accuracy vary across domains. Considering the knowledge domain as a moderator therefore helps clarify whether the educational contribution of GAI is context-sensitive rather than uniform across higher education settings.

Taken together, these moderators are not treated as purely exploratory statistical factors. Rather, they are grounded in the assumption that the effects of GAI depend on what the tool is used for, how long it is used, and the type of learning context in which it is embedded.

These conceptual and methodological limitations make a more focused synthesis necessary. In particular, higher education research still lacks a domain-specific meta-analytic account of whether GAI differentially affects intellectual and social–emotional outcomes under varying instructional conditions. Addressing this gap is important for both theory development and evidence-informed educational practice.

Based on the foregoing background, this study conducts a meta-analysis of the effects of GAI on IO and SO in higher education, based on a systematic literature search and structured study selection process. More specifically, the study has four objectives. First, it aims to quantify the overall effects of GAI on IO and SO separately. Second, it seeks to assess publication bias and examine the robustness of the pooled effects. Third, it investigates whether several study characteristics moderate the effects of GAI, including functional type, intervention duration, and the knowledge domain of the application context. Fourth, it further tests whether the overall effect differs significantly between IO and SO. Through these analyses, the present study aims to provide more nuanced and targeted evidence for understanding the educational role of GAI in higher education.

## 2 Materials and methods

### 2.1 Registration and protocol

This meta-analysis was registered in the International Prospective Register of Systematic Reviews (PROSPERO), an international database for systematic reviews in health and related fields, under registration number CRD420261361622 (Schiavo, 2019). The protocol was developed based on the PRISMA 2020 statement for complete, accurate, and transparent reporting (Page et al., 2021). No major deviations from the registered protocol occurred during the conduct of the review. Minor refinements were made to improve the clarity of variable classification and reporting structure in the final manuscript, but these changes did not alter the core research questions, eligibility criteria, the primary analytic strategy, or the overall review objectives.

### 2.2 Literature search

A systematic literature search was performed across three electronic databases: Web of Science Core Collection, Scopus, and the ERIC Database. These databases were chosen because they offer broad and complementary coverage of higher education, educational technology, psychology, and interdisciplinary scholarship related to GAI. The search encompassed the period from database inception to 26 January 2026. In addition, the reference lists of relevant review articles and all eligible full-text studies were manually examined to identify any potentially overlooked records.

The search strategy combined four concept blocks, namely, GAI, higher education, target outcomes, and experimental study design. Synonyms within each concept block were linked with OR, and the four blocks were combined with AND. The core search strategy was developed with reference to current recommendations for systematic review reporting and meta-analytic synthesis (Chandler et al., 2019). The core search strategy was as follows:

(“generative artificial intelligence” OR “generative AI” OR GenAI OR “large language model*” OR LLM OR ChatGPT OR GPT OR “GPT 3” OR “GPT 3.5” OR “GPT 4” OR “AI chatbot*” OR “conversational AI” OR Bard OR Gemini OR Claude OR Copilot)

AND (“higher education” OR “tertiary education” OR universit* OR college* OR undergraduate* OR postgraduate* OR “graduate student*” OR “university student*” OR “college student*”)

AND (“intellectual outcome*” OR “cognitive outcome*” OR “learning outcome*” OR “academic achievement” OR “academic performance” OR “learning performance” OR “knowledge acquisition” OR “knowledge gain” OR “learning achievement” OR “critical thinking” OR “problem solving” OR “writing performance” OR “social emotional outcome*” OR socioemotional OR “affective outcome*” OR motivation OR engagement OR “self efficacy” OR attitude OR perception OR satisfaction OR confidence OR anxiety) AND (random* OR experiment* OR quasi experiment* OR quasi experimental OR intervention* OR “controlled trial” OR “pretest posttest”)

Database-specific field tags and indexing terms were adjusted where necessary, but the conceptual structure of the search remained unchanged across databases.

### 2.3 Eligibility criteria

Studies were included only if they met all of the following criteria. First, the participants had to be students in higher education, including those enrolled in universities, colleges, and other tertiary institutions. Second, the intervention had to involve GAI or closely related large language model tools, with a clear application in teaching or learning contexts. Third, the study had to report at least one eligible outcome measure classified as either an intellectual outcome or a social–emotional outcome. Fourth, the study had to employ an experimental, quasi-experimental, or controlled comparative design. Fifth, sufficient quantitative data had to be available for effect size calculation, including the sample sizes of the intervention and comparison groups, as well as post-intervention means and standard deviations.

Studies were excluded if they met any of the following conditions. The participants were not higher education students. The study did not treat GAI as the core intervention. The study lacked a comparative condition. The required outcome measures were not reported. Insufficient quantitative data were provided for effect size estimation. In addition, reviews, theoretical papers, editorials, commentaries, study protocols, theses, conference abstracts from which usable outcome data could not be extracted, and purely qualitative studies were excluded. The formulation of these criteria was guided by the general principles of systematic reviews and meta-analyses.

### 2.4 Literature screening

All retrieved records were imported into EndNote 21 for reference management, and duplicates were removed before screening. Study selection was conducted in two sequential stages. In the first stage, titles and abstracts were screened to remove clearly ineligible records. Articles were excluded at this stage if it was not involve higher education participants (*n* = 365; e.g., Al Zakwani and Tamilarasi, 2026), did not examine a GAI-based educational intervention (*n* = 466; e.g., Al-Zubaidi et al., 2024), were not experimental or quasi-experimental primary studies (*n* = 269; e.g., Behnam and Ali, 2024), or did not report outcomes relevant to the present review framework, namely, intellectual or social–emotional outcomes (*n* = 352; e.g., Antoine et al., 2026).

In the second stage, the full texts of potentially eligible studies were reviewed against the prespecified inclusion and exclusion criteria. Full-text articles were excluded because of incompatible study design, ineligible study population, insufficient quantitative data for effect size extraction, or lack of a control group.

The entire screening procedure was conducted independently by two reviewers. Any disagreements were resolved through discussion, and a third reviewer was consulted when consensus could not be reached. The whole screening process is reported using the PRISMA flowchart.

### 2.5 Methodological quality assessment

The methodological quality of the included studies was assessed using the Mixed Methods Appraisal Tool (MMAT version 2018). This tool was selected because the present review included both randomized and non-randomized quantitative studies. Each study was first categorized according to the relevant MMAT design domain, and the corresponding five appraisal criteria were then applied. Two reviewers independently assessed the methodological quality of all included studies. Each criterion was rated as “Yes,” “No,” or “Cannot tell.” Any disagreements were resolved through discussion until a consensus was reached. In accordance with current guidance on MMAT use, the appraisal results were used to support the interpretation of the findings rather than to exclude studies from the meta-analysis.

### 2.6 Data extraction and coding

A structured data extraction form was developed before formal coding. Two reviewers independently extracted study-level information, and discrepancies were checked against the original articles and resolved by discussion. The following information was extracted from each eligible study: author and year of publication, sample size in the intervention and comparison groups, posttest means and standard deviations, functional type of GAI, outcome domain, outcome measure, intervention duration, and knowledge domain.

To enhance conceptual clarity, the outcome coding framework distinguished between intellectual outcomes and social–emotional outcomes on the basis of the primary function of the measured construct. Intellectual outcomes were defined as outcomes reflecting students’ cognitive, academic, or task-performance development in higher education. These included indicators such as academic achievement, knowledge acquisition, writing performance, feedback quality, problem-solving, critical thinking, and discipline-specific skill performance. Social–emotional outcomes were defined as outcomes reflecting students’ affective, motivational, or self-perceptual responses to learning. These included indicators such as motivation, self-efficacy, engagement, willingness to communicate, enjoyment, and anxiety.

In cases where constructs were potentially overlapping, classification was guided by their dominant theoretical orientation rather than by surface similarity alone. For example, self-efficacy and engagement were coded as social–emotional outcomes because they primarily reflect learners’ motivational beliefs, affective investment, and readiness to participate rather than direct task performance itself. By contrast, critical thinking was coded as an intellectual outcome because it reflects higher-order cognitive processing and performance-related reasoning. For negatively valenced constructs such as anxiety, effect directions were coded so that positive effect sizes consistently reflected more favorable social–emotional functioning. When a study reported multiple outcomes within the same domain, coding decisions were made according to a prespecified hierarchy, prioritizing the outcome most conceptually central to the study aim or the most direct indicator of the target domain.

Outcomes were coded into two overarching domains. IO included academic achievement, learning performance, knowledge gain, writing performance, critical thinking, problem-solving, and other conceptually related cognitive indicators. SO included motivation, engagement, self-efficacy, attitudes, satisfaction, confidence, anxiety, and other affective or socioemotional indicators. To preserve the independence of effect sizes, one eligible posttest effect size was retained for each study within the coded outcome domain.

Functional types of GAI were classified according to their principal educational role. For IO, functional types were coded as assessment, personalized learning support, and personalized tutoring systems. For SO, the original categories were consolidated into two broader groups, namely, personalized learning support and structured systems, because some original subgroups contained few studies and the combined category represented tools with more structured instructional or evaluative functions.

Intervention duration was also coded for moderator analyses. For IO, duration was grouped into less than or equal to 4 weeks, greater than 4–8 weeks, and more than 8 weeks. For SO, duration was regrouped into less than or equal to 4 weeks and more than 4 weeks because the number of studies in the longest duration category was too small to support a stable three-group comparison.

Knowledge domains were coded to reflect the disciplinary context of each intervention. For IO, the original disciplinary labels were reorganized into three broader categories, namely, Applied and Technical Disciplines, Arts and Humanities, and Education and Humanities, to improve interpretability while maintaining adequate numbers of studies in each subgroup. For SO, knowledge domain was recorded descriptively but was not used as a primary moderator because the distribution of studies across categories was too sparse for a stable formal subgroup analysis.

To address potential non-independence, only one effect size per study was retained for the quantitative synthesis unless the study clearly contributed to a distinct prespecified domain-specific analysis. When a study reported both intellectual outcomes and social–emotional outcomes, we avoided entering multiple correlated effects from the same sample into the same pooled dataset. Instead, one eligible effect size was retained according to a prespecified decision rule based on conceptual centrality, reporting completeness, and comparability with the broader evidence base. For example, some included studies reported both performance-related and affective outcomes, such as creative problem-solving performance together with self-efficacy, or learning effectiveness together with flow-related measures (e.g., Urban et al., 2024; Shi et al., 2024). In such cases, only one domain-specific effect size was retained for the main synthesis to preserve statistical independence.

When a study reported several outcomes within the same intellectual domain, such as multiple academic or performance indicators, we retained the outcome that was most conceptually central to the study’s aim and most comparable to other included studies. If an overall score was available alongside several subdimensions, the overall score was prioritized over individual subscales to avoid overrepresenting a single sample. For example, studies such as Shi et al. (2024) reported both an overall learning effectiveness measure and multiple subdimensions, whereas Urban et al. (2024) reported several related performance indicators. In these situations, one effect size was retained rather than entering multiple correlated outcomes from the same participants.

With regard to multiple intervention groups or multiple comparison groups, no included study contributed more than one eligible intervention arm and one eligible comparison arm to the final quantitative synthesis after the outcome selection rules were applied. The majority of included studies used a standard two-group structure consisting of a single intervention group and a single comparison group. Nevertheless, a study included multiple eligible intervention or comparison groups, conceptually similar groups would have been combined, or the shared comparison group would have been divided, in accordance with standard meta-analytic practice, to avoid double-counting.

When several posttests or repeated observations were reported, only the post-intervention assessment corresponding most closely to the end of the eligible intervention period was retained for effect size calculation. This rule was adopted to maximize comparability across studies and to prevent dependence caused by repeated measurements from the same sample. For longitudinal or repeated-measures studies, only one endpoint was therefore entered into the meta-analysis. The retained outcome used for independent effect size extraction for each included study is reported in Supplementary Table 3.

### 2.7 Statistical analysis

All statistical analyses were performed using Stata version 18. Effect sizes were calculated as Hedges’ g based on the posttest means, standard deviations, and sample sizes of the intervention and comparison groups (Hedges, 1981). Positive values indicated more favorable outcomes in the GAI group than in the comparison group.

Given the expected variation across educational settings, intervention formats, outcome measures, and disciplinary contexts, all pooled analyses were conducted using random-effects models estimated with restricted maximum likelihood. For each synthesis, the pooled effect size, 95% confidence interval, z-statistic, and *p*-value were calculated. Statistical heterogeneity was assessed using Cochran’s Q, tau-squared, and I-squared. Separate meta-analyses were conducted for IO and SO. Moderator analyses were performed by random-effects meta-regression and subgroup synthesis. For IO, moderator analyses examined functional types of GAI, intervention duration, and knowledge domains. For SO, moderator analyses examined functional types of GAI and intervention duration. An additional meta-regression was conducted to test whether outcome domain, namely, intellectual and SO, significantly moderated the pooled effect across all included studies.

Publication bias was assessed through visual inspection of funnel plots and by Egger’s regression test and Begg’s rank correlation test when the number of studies in a synthesis was sufficient for formal assessment (Begg and Mazumdar, 1994). Sensitivity analysis was conducted using a leave-one-out approach, in which each study was omitted to evaluate the robustness of the pooled estimate. The interpretation of publication bias tests was undertaken cautiously because funnel plot asymmetry may also reflect small-study effects and heterogeneity rather than publication bias alone (Egger et al., 1997).

Compared with the registered protocol, the final manuscript included clearer conceptual differentiation between intellectual outcomes and social–emotional outcomes and a more explicit presentation of moderator coding categories. These refinements were introduced to improve interpretability and reporting transparency rather than to change the substantive design of the review.

## 3 Results

### 3.1 Study selection and characteristics

**Figure 1:**
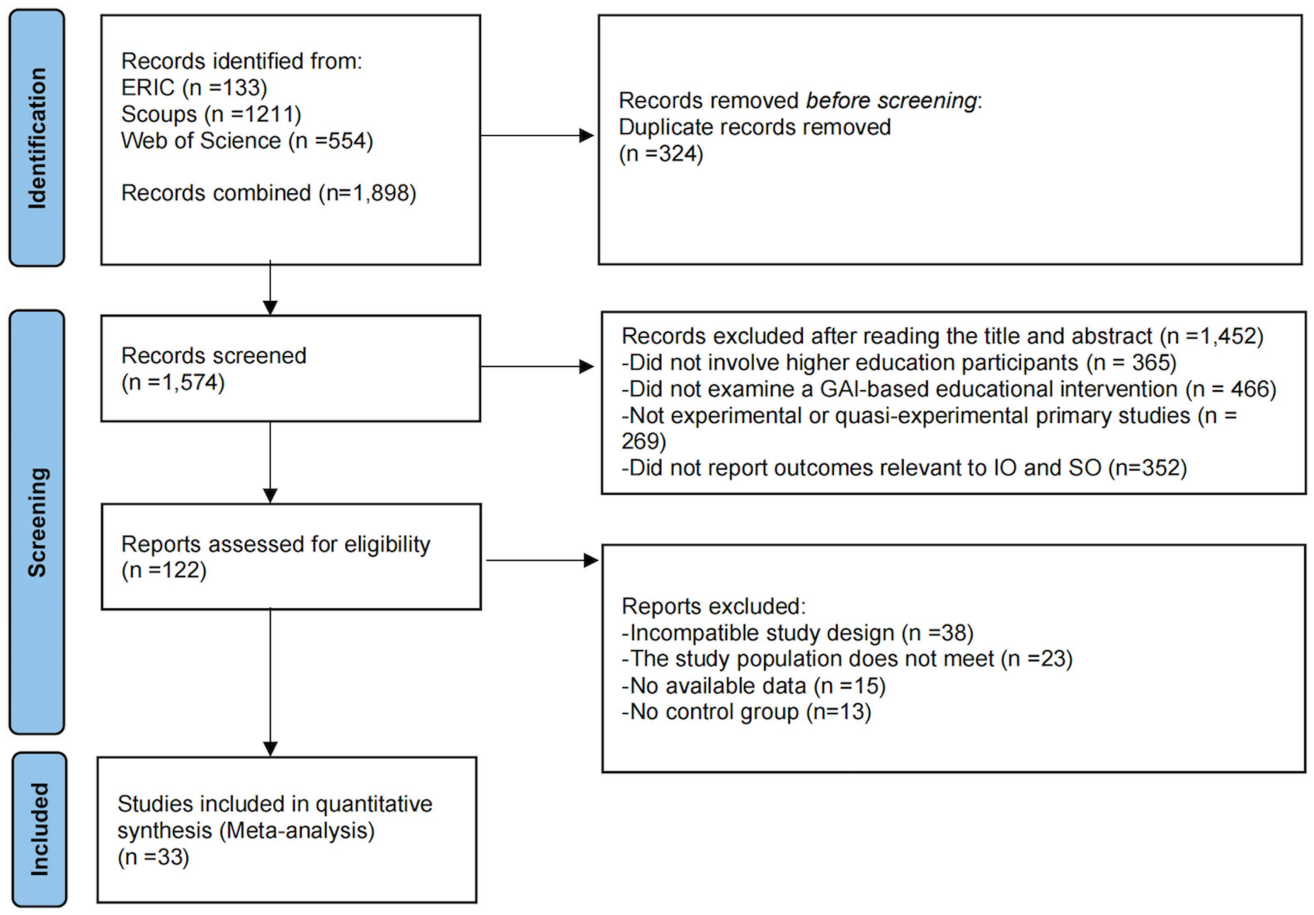
PRISMA flow diagram of study selection.

The study selection process is presented in Figure 1. A total of 1,898 records were initially identified from three electronic databases, namely, ERIC with 133 records, Scopus with 1,211 records, and Web of Science with 554 records. After the removal of 324 duplicate records, 1,574 records remained for title and abstract screening. Of these, 1,452 records were excluded as clearly irrelevant. As a result, 122 full-text reports were assessed for eligibility. Following full-text review, 89 reports were excluded for the following reasons: incompatible study design, 38; ineligible study population, 23; unavailable quantitative data, 15; and absence of a control group, 13. Ultimately, 33 studies met the eligibility criteria and were included in the quantitative synthesis (Supplementary data file).

The main characteristics of the included studies are summarized in Supplementary Table 2 with 33 studies (Yin et al., 2021; Vázquez-Cano et al., 2021; Mirzababaei and Pammer-Schindler, 2022; Kim and Lee, 2023; Kuo and Chen, 2023; Lee et al., 2022; Essel et al., 2022; Han et al., 2022; Escalante et al., 2023; Yilmaz and Yilmaz, 2023; Al Al Kahf et al., 2023; Li, 2023; Liu et al., 2024; Zhang R. et al., 2024; Hakiki et al., 2023; Silitonga et al., 2023; Qureshi, 2023; Sadek and Mohamed, 2023; Pellas, 2023; Song and Song, 2023; Li et al., 2025; Saritepeci and Durak, 2024; Wang and Xue, 2024; Yin et al., 2024; Bhatia et al., 2024; Chen and Hou, 2024; Essel et al., 2024; Guo et al., 2024; Boudouaia et al., 2024; Fathi et al., 2024; Zhang C. et al., 2024; Shi et al., 2024; Urban et al., 2024). The 33 studies were published between 2021 and 2024, and the number of publications increased markedly over time. Specifically, 2 studies were published in 2021, 6 in 2022, 12 in 2023, and 13 in 2024. The included studies were conducted across a broad range of countries and regions, indicating growing international interest in the use of GAI in higher education. China contributed the largest number of studies, followed by Turkey, Ghana, Indonesia, and several studies from Europe, the Middle East, and other parts of Asia and Africa.

As shown in Supplementary Table 2, the included studies were methodologically diverse, although quasi-experimental and pretest-posttest designs predominated, with only one randomized controlled trial identified. The overall sample was large, with 3,394 participants across intervention and control groups. In terms of intervention characteristics, the studies mainly examined GAI as personalized learning support, personalized tutoring systems, or assessment-oriented tools, with personalized learning support being the most common. More studies focused on intellectual outcomes than on social–emotional outcomes. The intervention duration and disciplinary contexts also varied although the evidence base was concentrated primarily in arts and humanities, education, and information and communication technologies. Overall, the included studies indicate a heterogeneous but rapidly developing evidence base, while the full descriptive breakdown is provided in Supplementary Table 2.

Where studies reported multiple eligible outcomes, repeated assessments, or more than one relevant domain, one independent effect size was retained according to the prespecified decision rules described in Supplementary Table 3.

### 3.2 Overall effect on IO

A random-effects meta-analysis was used to evaluate the impact of GAI on IO in higher education. As shown in Supplementary Table 4, after a combined analysis of 21 studies, it was found that the overall effect showed a statistically significant positive effect. Hedges’ g was 1.096, and the 95% confidence interval was 0.087 to 2.104. The overall effect is statistically significant (z = 2.13, *p* = 0.0332). These results show that GAI is related to the significant positive improvement of IOs of higher education students.

**Figure 2:**
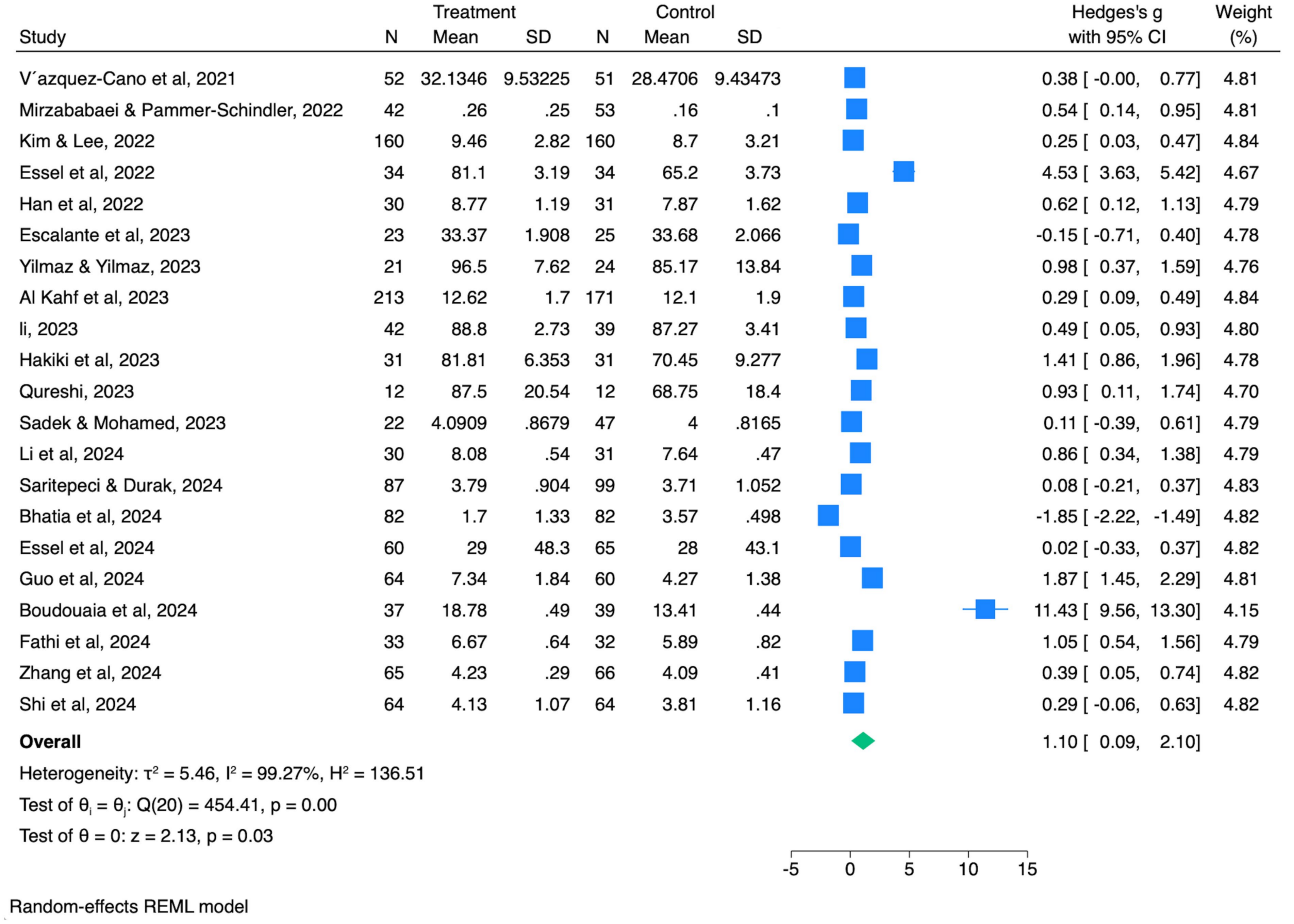
Forest plot of the overall effect on IO.

At the same time, the heterogeneity between studies is extremely high. Heterogeneity statistics show that tau-squared = 5.4588, I-squared = 99.27%, and H-squared = 136.51. In addition, the results of the Cochran *Q* test are highly significant (*Q* = 454.41, *p* < 0.001). These results show that there is a significant variation in the magnitude of the effect observed between different studies. Therefore, it is appropriate to use a random-effects model for effect volume consolidation. As shown in Figure 2, the forest plot further shows that the distribution of specific estimates in each study is relatively scattered. Several studies have reported very large positive effects, and at least one study has shown significant negative effects. This pattern is consistent with the extremely high heterogeneity results observed in the combined analysis.

### 3.3 Overall effect on SO

**Figure 3:**
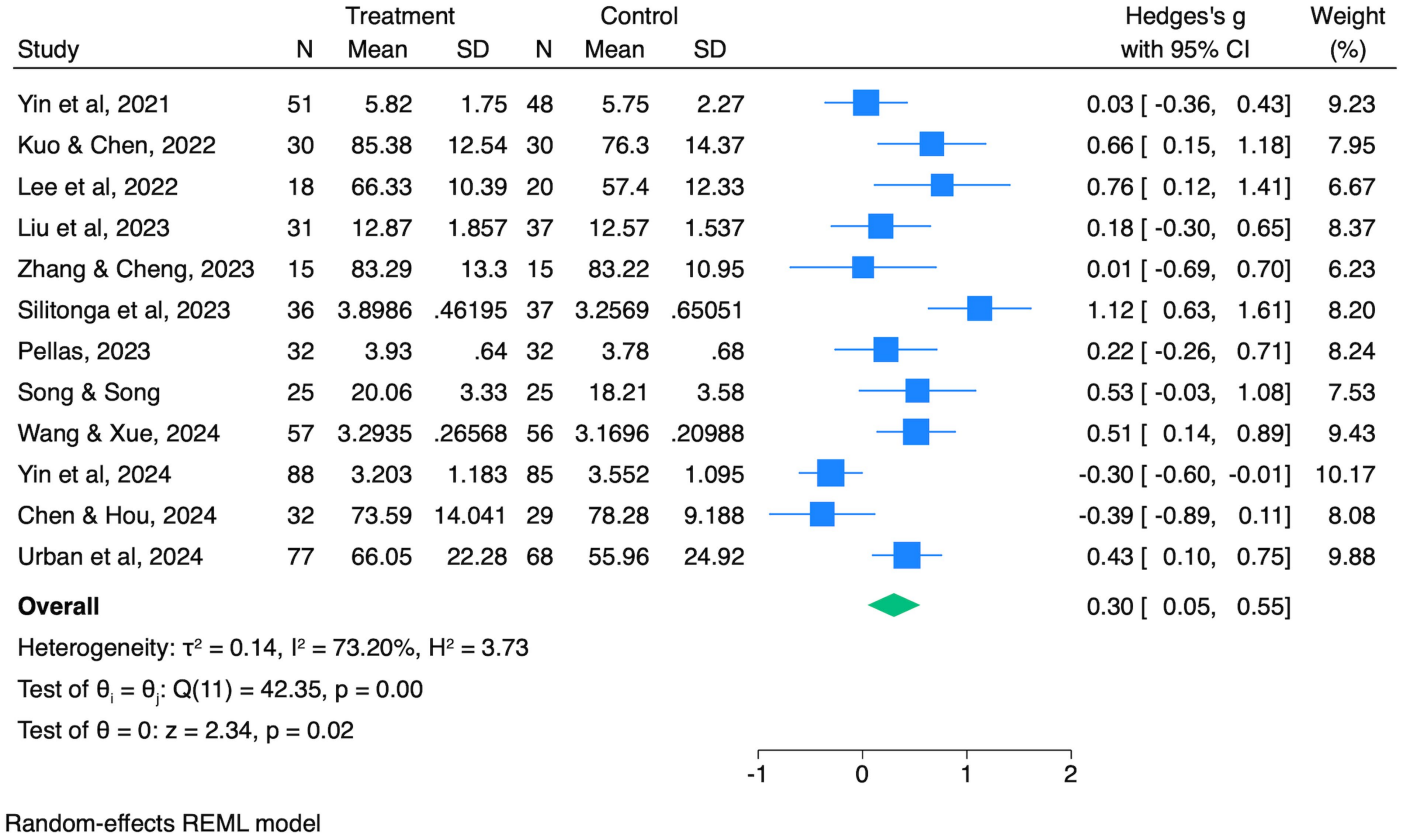
Forest plot of the overall effect on SO.

A random-effects meta-analysis was conducted to examine the effect of GAI on SO in higher education. As shown in Supplementary Table 4 and Figure 3, the pooled analysis of 12 studies showed a statistically significant positive effect, with a Hedges’ g of 0.301 and a 95% confidence interval ranging from 0.048 to 0.553. The overall effect was statistically significant, with z = 2.34 and *p* = 0.0195. These findings indicate that GAI has a small but statistically significant positive effect on the social–emotional outcomes of students in higher education.

High heterogeneity has been observed in each study. Heterogeneity statistics show that tau^2^ = 0.1395, *I*^2^ = 73.20%, and *H*^2^ = 3.73. In addition, Cochran’s Q test was statistically significant (*Q* = 42.35, *p* < 0.001), indicating considerable variability across the included studies. Given both the magnitude and statistical significance of the heterogeneity, the use of a random-effects model was considered appropriate for pooling the effect sizes.

### 3.4 Publication bias

Publication bias evaluation was carried out to judge whether the merger effect may be affected by small-study effects or selective publication. No obvious asymmetry was found in the visual inspection of the funnel plot, which indicates that the distribution of the effect is relatively balanced. To further evaluate this problem, Egger’s regression test and Begg’s rank correlation test were carried out at the same time.

#### 3.4.1 IO

**Figure 4:**
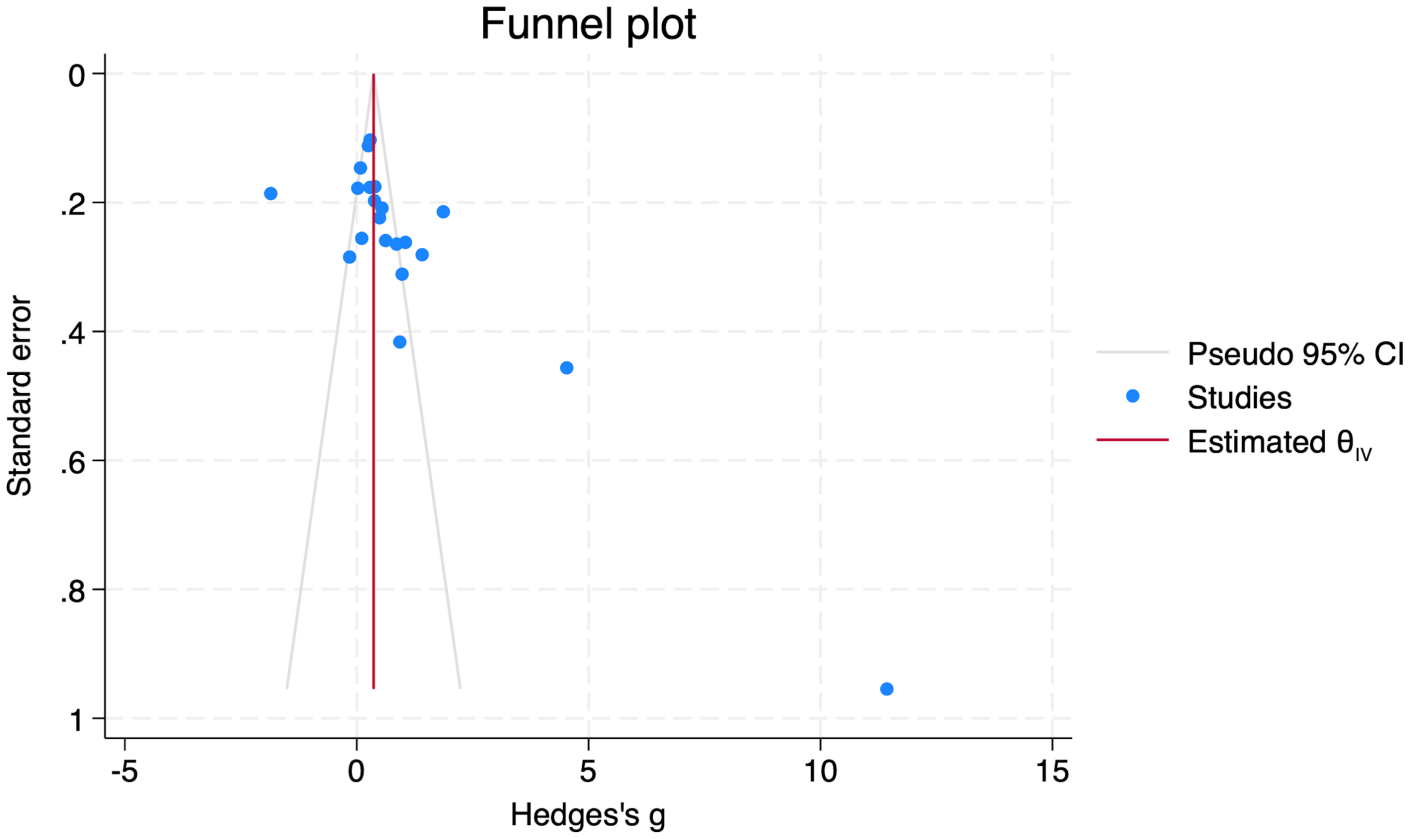
Funnel plot of publication bias on IO.

As reported in Supplementary Table 5, Egger’s regression test of IO indicated significant small-study effects, with beta1 = 12.60, SE = 1.530, z = 8.23, and *p* < 0.001. Begg’s rank correlation test produced a consistent result, with Kendall’s score = 110.00, SE = 33.116, z = 3.29, and *p* = 0.0010. Taken together with the asymmetrical appearance of the funnel plot (Figure 4), these findings suggest the presence of substantial publication bias or small-study effects in the studies examining IO. Therefore, the pooled effect for IO should be interpreted with caution.

#### 3.4.2 SO

**Figure 5:**
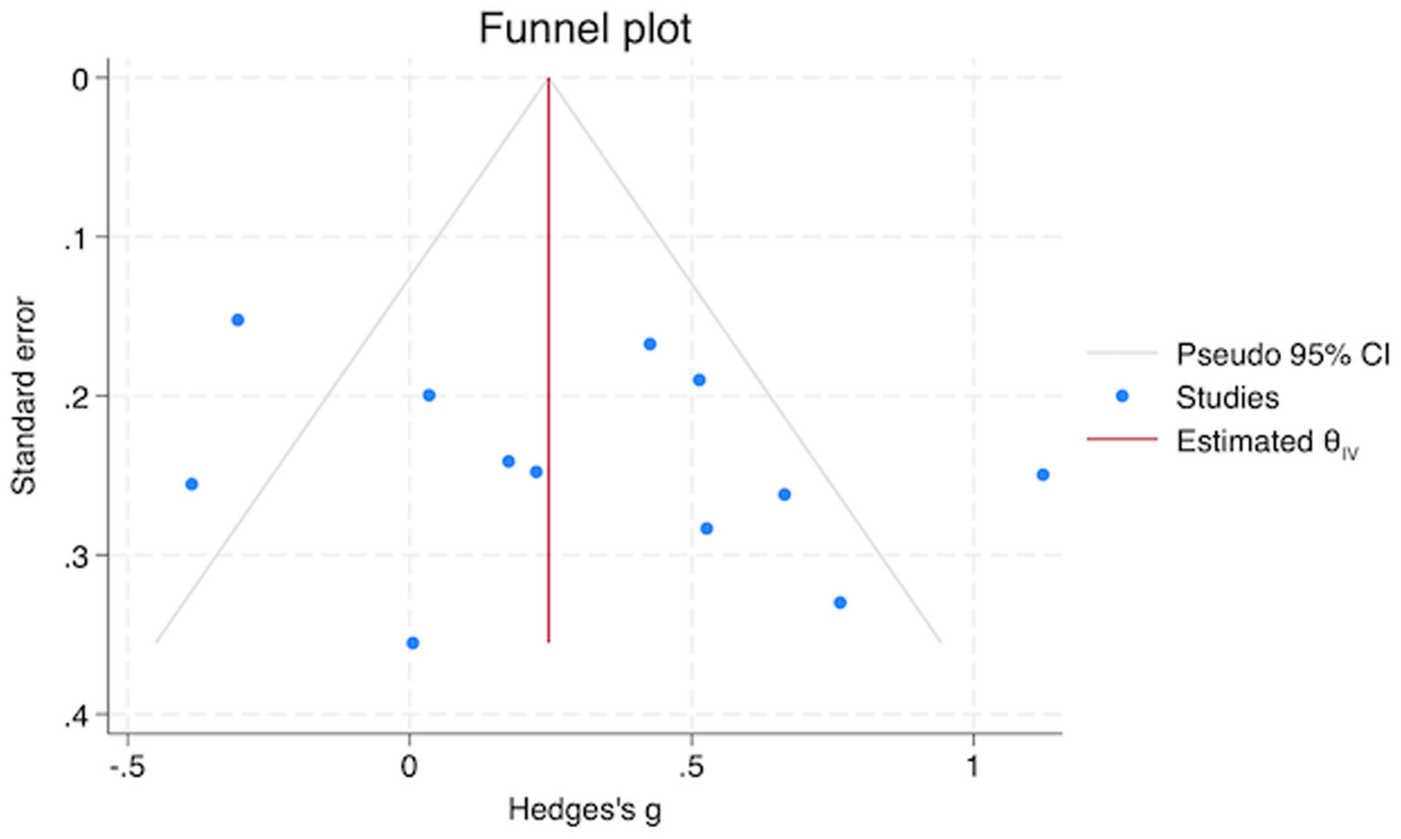
Funnel plot of publication bias on SO.

As reported in Supplementary Table 5, Egger’s regression test of SO showed no evidence of significant small-study effects, with beta1 = 1.92, SE = 2.260, z = 0.85, and *p* = 0.3967. Similarly, Begg’s rank correlation test was not statistically significant, with Kendall’s score = 12.00, SE = 14.583, z = 0.75, and *p* = 0.4507. Taken together with the visual appearance of the funnel plot (Figure 5), these findings suggest that there was no substantial evidence of publication bias in the studies examining SO.

### 3.5 Sensitivity analysis

Sensitivity analysis was performed to assess the robustness of the pooled estimates. A leave-one-out procedure was used, in which each study was sequentially omitted and the overall effect size was recalculated. This approach allows examination of whether the overall findings are unduly driven by any single study.

#### 3.5.1 Sensitivity analysis of IO

As shown in Supplementary Table 6, the leave-one-out sensitivity analysis showed that the pooled effect size for IO remained positive across all iterations, ranging from 0.625 to 1.242. In most cases, after deleting a single study, the combined effect remains statistically significant. However, the size of the combined estimate and its significance will change to some extent depending on the different studies deleted, indicating that several studies have had a more significant impact on the overall results.

When Boudouaia et al. (2024) is deleted, the decrease in the merger effect is the greatest. At this time, Hedges’ g is 0.625, and the 95% confidence interval is 0.140 to 1.110 (*p* = 0.011). After deleting Bhatia et al. (2024), the strongest consolidated estimate was observed. At this time, Hedges’ g is 1.242, and the 95% confidence interval is 0.229 to 2.255 (*p* = 0.016). It is worth noting that after deleting Essel et al. (2022), the merger effect is no longer statistically significant. Hedges’ g is 0.922, and 95% confidence interval is −0.067 to 1.910 (*p* = 0.068). Similarly, after deleting Guo et al. (2024), a marginally significant result was obtained. Hedges’ *g* is 1.060, and the 95% confidence interval ranges from −0.002 to 2.123 (*p* = 0. 050).

Overall, these results show that although the direction of the consolidation effect of IO remains positive, the size of the summary estimate and its significance are more sensitive to the deletion of some studies. This pattern is consistent with the extremely high heterogeneity and publication bias evidence observed in the IO dataset.

#### 3.5.2 Sensitivity analysis of SO

As shown in Supplementary Table 7, the leave-one-out sensitivity analysis indicated that the pooled effect size for SO remained positive across all iterations, ranging from 0.222 to 0.369. In most cases, the statistical significance of the pooled effect was preserved after the omission of a single study. The largest attenuation was observed when Silitonga et al. (2023) was excluded, yielding Hedges’ g = 0.222, 95% CI from −0.004 to 0.448, and *p* = 0.054. Although this result was marginally non-significant, the magnitude and direction of the pooled estimate remained broadly consistent with the main analysis. Exclusion of all other studies resulted in pooled effects that remained statistically significant. Overall, these findings indicate that the association between GAI and SO was generally stable and was not driven by any single study to a substantial extent.

### 3.6 Moderator analyses for IO

#### 3.6.1 Functional types of GAI

To examine whether the effect of GAI on IO varied according to functional type, a random-effects meta-regression was conducted with functional type entered as a categorical moderator. The omnibus test showed that functional type did not significantly moderate the pooled effect on IO, with Wald chi-square = 1.10 and *p* = 0.5759. The proportion of between-study variance explained by this moderator was negligible, as indicated by R-squared = 0.00%. Residual heterogeneity remained extremely high after inclusion of the moderator, with residual tau-squared = 5.871 and residual I-squared = 99.27%.

Using assessment as the reference category, the regression coefficient for personalized learning support was −0.805, with a standard error of 1.303, z = −0.62, *p* = 0.537, and a 95% confidence interval from −3.358 to 1.748. The regression coefficient for personalized tutoring systems was 0.561, with a standard error of 1.308, z = 0.43, *p* = 0.668, and a 95% confidence interval from −2.004 to 3.125. These findings indicate that neither personalized learning support nor personalized tutoring systems differed significantly from assessment in terms of their pooled effects on IO.

As reported in Supplementary Table 8, subgroup analyses further showed that the assessment category yielded a statistically significant positive effect on IO, with Hedges’ *g* = 1.168 and a 95% confidence interval from 0.019 to 2.316. However, heterogeneity within this subgroup remained very high, with I-squared = 98.03%. Personalized learning support also produced a significant positive effect, with Hedges’ *g* = 0.291 and a 95% confidence interval from 0.099 to 0.483, accompanied by moderate heterogeneity, *I*-squared = 47.16%. By contrast, personalized tutoring systems showed the largest numerical pooled effect, Hedges’ *g* = 1.858, but this effect did not reach statistical significance, with a 95% confidence interval from −1.242 to 4.958 and I-squared = 99.67%.

Overall, although the magnitude of pooled effects varied across functional categories, the between-group difference was not statistically significant. Therefore, the present findings do not support functional type as a significant moderator of the relationship between GAI and IO in higher education.

#### 3.6.2 Intervention duration

To examine whether the effect of GAI on IO varied according to intervention duration, a random-effects meta-regression was performed with intervention duration entered as a categorical moderator. The omnibus test indicated that intervention duration did not reach conventional statistical significance as a moderator of the pooled effect on IO, with Wald chi-square = 4.96 and *p* = 0.0836. Nevertheless, the moderator accounted for a modest proportion of between-study variance, with *R*-squared = 12.34%. Considerable residual heterogeneity remained after inclusion of the moderator, with residual tau-squared = 4.785 and residual *I*-squared = 99.09%.

Using less than or equal to 4 weeks as the reference category, the regression coefficient for greater than 4 to 8 weeks was 0.113, with a standard error of 1.227, z = 0.09, *p* = 0.927, and a 95% confidence interval from −2.293 to 2.518. This result shows that there is no significant difference between short-term intervention and medium-term intervention. On the contrary, the regression coefficient of the intervention duration of more than 8 weeks is 2.327, the standard error is 1.117, z = 2.08, *p* = 0.037, and the 95% confidence interval is 0.138 to 4.516, which indicates that longer interventions are associated with significantly larger effect sizes than interventions that last no more than 4 weeks.

As shown in Supplementary Table 9, subgroup analyses further provide descriptive evidence of the difference in the duration of the intervention. In the study with an intervention duration of no more than 4 weeks, the combined Hedges’ g was 0.281, and the 95% confidence interval was −0.314 to 0.876, and the effect was not statistically significant. Studies with intervention durations greater than 4 to 8 weeks showed a pooled Hedges’ g of 0.378, with a 95% confidence interval from −0.007 to 0.764, which was also not statistically significant at the conventional threshold. Studies with intervention durations longer than 8 weeks produced the largest pooled effect with Hedges’ g = 2.755, but this estimate remained non-significant because of its very wide confidence interval from −0.181 to 5.692. The heterogeneity of this subgroup was extremely high, with *I*-squared = 99.58%.

Overall, these results show that intervention duration may tend to affect the effectiveness of GAI on IO, especially when the intervention lasts more than 8 weeks. However, since the overall omnibus test has not reached statistical significance and there is still a large residual heterogeneity, the regulatory effect of intervention duration should be explained carefully.

#### 3.6.3 Knowledge domains

To further examine whether the effect of GAI on IO varied across disciplinary contexts, a random-effects meta-regression was conducted with knowledge domains entered as a categorical moderator. The omnibus test indicated that knowledge domain did not significantly moderate the pooled effect on IO, with Wald chi-square = 3.88 and *p* = 0.1439. The proportion of between-study variance explained by this moderator was limited, as reflected by R-squared = 6.18%. Moreover, substantial residual heterogeneity remained after the inclusion of knowledge domain, with residual tau-squared = 5.122 and residual *I*-squared = 99.20%.

Using Applied and Technical Disciplines as the reference category, the regression coefficient for Arts and Humanities was 2.386, with a standard error of 1.412, z = 1.69, *p* = 0.091, and a 95% confidence interval from −0.381 to 5.153. The regression coefficient for Education and Humanities was −0.237, with a standard error of 1.107, z = −0.21, *p* = 0.830, and a 95% confidence interval from −2.406 to 1.932. These results indicate that neither Arts and Humanities nor Education and Humanities differed significantly from Applied and Technical Disciplines in terms of their pooled effects on IO.

As shown in Supplementary Table 10, subgroup analyses provide further descriptive information. The combined Hedges’ g of the Applied and Technical Disciplines subgroup is 0.741, the 95% confidence interval is minus 0.460 to 1.942, and the effect is not statistically significant. The Arts and Humanities subgroup shows the largest numerical consolidation effect, with Hedges’ g = 3.372, but because its confidence interval is very wide, it is −1.781 to 8.524. Therefore, the estimate also does not reach statistical significance. In contrast, the Education and Humanities subgroup showed a statistically significant positive effect, with Hedges’ g = 0.505 and a 95% confidence interval from 0.071 to 0.940. Nevertheless, heterogeneity remained substantial across all subgroups, indicating marked between-study variability within each disciplinary category.

Taken together, these findings suggest that although the magnitude of pooled effects varied across knowledge domains, the between-group difference did not reach statistical significance. Therefore, the current evidence does not support knowledge domain as a significant moderator of the relationship between GAI and IO in higher education.

### 3.7 Moderator analyses for SO

#### 3.7.1 Functional types of GAI

To further examine whether the effect of GAI on SO varied according to functional type, a random-effects meta-regression was conducted with functional type entered as a categorical moderator. In the present analysis, functional types were grouped into personalized learning support and structured systems. The omnibus test indicated that functional type did not significantly moderate the pooled effect on SO, with Wald chi-square = 0.19 and *p* = 0.6601. The proportion of between-study variance explained by this moderator was negligible, with R-squared = 0.00%. In addition, considerable residual heterogeneity remained after inclusion of the moderator, with residual tau-squared = 0.1557 and residual I-squared = 74.71%.

Using personalized learning support as the reference category, the regression coefficient for structured systems was 0.119, with a standard error of 0.271, z = 0.44, p = 0.660, and a 95% confidence interval from −0.412 to 0.650. This result shows that there is no significant difference between structured systems and personalized learning support in terms of the combined effect on SO.

As shown in Supplementary Table 11, subgroup analyses provide further descriptive evidence. The combined Hedges’ *g* of the personalized learning support subgroup is 0.245, the 95% confidence interval is −0.017 to 0.507, and the effect has not reached statistical significance. The heterogeneity of this subgroup was moderate, with *I*-squared = 52.83%. The structured systems subgroup showed a numerically larger pooled effect, with Hedges’ *g* = 0.377, but this estimate was similarly not statistically significant, with a 95% confidence interval from −0.123 to 0.877. The heterogeneity within this subgroup was high, with *I*-squared = 85.20%.

Taken together, these findings suggest that although the pooled effect size for structured systems was numerically larger than that for personalized learning support, the between-group difference was not statistically significant. Therefore, the current evidence does not support functional type as a significant moderator of the relationship between GAI and SO in higher education.

#### 3.7.2 Intervention duration

To further examine whether the effect of GAI on SO varied according to intervention duration, a random-effects meta-regression was conducted with intervention duration entered as a categorical moderator. In the present analysis, intervention duration was grouped into less than or equal to 4 weeks and more than 4 weeks. The omnibus test indicated that intervention duration did not significantly moderate the pooled effect on SO, with Wald chi-square = 0.00 and *p* = 0.9572. The proportion of between-study variance explained by this moderator was negligible, with *R*-squared = 0.00%. In addition, substantial residual heterogeneity remained after inclusion of the moderator, with residual tau-squared = 0.158 and residual *I*-squared = 75.64%.

Using less than or equal to 4 weeks as the reference category, the regression coefficient for more than 4 weeks was 0.015, with a standard error of 0.277, z = 0.05, p = 0.957, and a 95% confidence interval from −0.527 to 0.557. This result indicates that studies with longer intervention duration did not differ significantly from shorter interventions in terms of their pooled effects on SO.

As reported in Supplementary Table 12, subgroup analyses provided further descriptive evidence. The subgroup with intervention durations of less than or equal to 4 weeks yielded a pooled Hedges’ g of 0.286, with a 95% confidence interval from 0.000 to 0.572, and this effect reached statistical significance. The heterogeneity of this subgroup was substantial, with I-squared = 70.73%. The subgroup with intervention durations of more than 4 weeks showed a pooled Hedges’ *g* of 0.307, with a 95% confidence interval from −0.206 to 0.821, and this effect did not reach statistical significance. The heterogeneity within this subgroup was also substantial, with *I*-squared = 78.09%.

Taken together, these findings suggest that the pooled effect sizes for shorter and longer interventions were highly similar in magnitude, and the between-group difference was not statistically significant. Therefore, the current evidence does not support intervention duration as a significant moderator of the relationship between GAI and SO in higher education.

### 3.8 Outcome domain as a moderator

To further examine whether the effectiveness of GAI differed across outcome domains, a random-effects meta-regression was conducted with outcome domain entered as a categorical moderator. The pooled analyses showed that GAI had a statistically significant positive effect on both IO and SO in higher education. As reported in Supplementary Table 13, the pooled effect for IO was 1.096, with a 95% confidence interval from 0.087 to 2.104, whereas the pooled effect for SO was 0.301, with a 95% confidence interval from 0.048 to 0.553.

Although the pooled effect size was numerically larger for IO than for SO, the moderator analysis did not indicate a statistically significant difference between the two domains. With IO specified as the reference category, the regression coefficient for SO was −0.739, with a standard error of 0.645, z = −1.15, *p* = 0.252, and a 95% confidence interval from −2.004 to 0.526. The omnibus test of moderation was similarly not significant, with Wald chi-square = 1.31 and *p* = 0.2522, as reported in Supplementary Table 14.

These findings suggest that, although GAI appeared to show a stronger pooled association with IO than with SO, this difference was not statistically reliable. In addition, substantial residual heterogeneity remained after inclusion of the outcome domain as a moderator, with residual tau^2^ = 3.105 and residual I^2^ = 98.62%. The explained proportion of between-study variance was 0.00%, indicating that the outcome domain did not account for the considerable heterogeneity observed across studies. Therefore, the distinction between intellectual and SO did not significantly moderate the overall effectiveness of GAI in higher education.

### 3.9 Methodological quality assessment

The methodological quality appraisal results are presented in Supplementary Table 15. Of the 33 included studies, 7 were classified as randomized studies and 26 as non-randomized studies according to the MMAT framework. Overall, the methodological quality of the included studies was acceptable although variations in reporting and design rigor were evident.

Among the randomized studies, baseline comparability and intervention adherence were generally satisfactory, whereas the reporting of randomization procedures and blinding of outcome assessors was often insufficient. Among the non-randomized studies, the criteria related to the appropriateness of measurements, completeness of outcome data, and intervention implementation were most frequently satisfied. However, participant representativeness was often unclear, and the extent to which potential confounders were adequately controlled varied across studies.

Taken together, these findings suggest that the current evidence base is methodologically adequate for quantitative synthesis, but the pooled findings should still be interpreted with caution in light of the variability in study design and reporting quality.

## 4 Discussion

This systematic review and meta-analysis synthesized evidence from 33 experimental and quasi-experimental studies to investigate the effects of GAI on IO and SO in higher education. Overall, the findings indicated that GAI yielded statistically significant positive effects across both outcome domains. The pooled effect size was numerically larger for IO than for SO, although the direct comparison between the two domains did not achieve statistical significance. Publication bias and sensitivity analyses further suggested that the evidence base for IO was less stable and exhibited greater heterogeneity than that for SO, whereas moderator analyses showed that functional type, intervention duration, and knowledge domain did not consistently account for between-study variation at the omnibus level.

### 4.1 Comparison with previous literature

One of the central findings of the present study is that GAI had a significant positive effect on IO in higher education. This result is broadly consistent with recent meta-analyses and systematic reviews showing that ChatGPT and related GAI tools can improve academic achievement, learning performance, writing-related performance, and higher-order learning outcomes when they are integrated into instructional tasks in a purposeful way (Oliński and Krukowski, 2025). Existing evidence has repeatedly suggested that these tools are most effective when they function as immediate sources of explanation, feedback, task support, and cognitive scaffolding (Radhwan, 2025). From this perspective, the present findings reinforce the growing view that GAI can enhance students’ cognitive performance not simply because it provides answers quickly, but because it can reduce access barriers to information, externalize examples, prompt revision, and support iterative engagement with academic tasks. At the same time, this synthesis adds specificity by focusing only on higher education and by separating IO from SO rather than collapsing all outcomes into a single broad indicator. This distinction is important because it shows that the strongest and most consistent benefits of GAI still appear to lie in directly performance-related domains.

The present study also found a smaller but still significant overall effect for SO. This result is similarly in line with previous reviews showing that GAI can support students’ motivation, confidence, engagement, and perceived usefulness; however, such effects are generally less stable and more context-dependent than cognitive gains (Harjanto et al., 2025). Prior work has emphasized that students often appreciate the immediacy, availability, and personalization of GAI, especially when they perceive it as a non-judgmental and responsive learning companion (Grassini et al., 2024). However, the same body of literature also identifies major concerns, including overreliance, reduced independent effort, misinformation, academic integrity anxiety, and uncertainty regarding appropriate classroom integration (Milakis et al., 2025). The present findings fit well with this more nuanced view. Social–emotional benefits were present, but they were modest in size and were not systematically moderated by the major design features examined in this study (Wang et al., 2024). This suggests that motivational and affective outcomes may depend less on the technology alone and more on how that technology is pedagogically framed, socially legitimized, and aligned with course expectations. In other words, while GAI can support students emotionally and motivationally, its influence in this domain appears to be more contingent and less automatic than its influence on task-related intellectual performance.

A further point of connection with the previous literature concerns the absence of strong omnibus moderator effects. In the IO analyses, neither functional type, intervention duration, nor knowledge domain produced a statistically significant omnibus moderator effect even though certain subgroup patterns appeared noteworthy. Similarly, in the SO analyses, neither functional type nor intervention duration emerged as a statistically significant moderator. This general pattern is compatible with the broader state of the field. Current empirical research on GAI in education remains highly heterogeneous in terms of conceptualization, task design, teacher guidance, learner autonomy, and disciplinary context. Outcome measurement also varies substantially across studies. Therefore, many differences that seem meaningful at the descriptive level may show statistical instability when tested in an evidence base that is still limited and unevenly distributed. This may explain why although some promising subgroup trends were observed in this study, strong and consistent moderator effects were not found across the complete dataset.

The findings of this review should also be interpreted in light of the methodological quality of the included studies. The MMAT appraisal suggested that the overall quality of the evidence base was acceptable, particularly with respect to measurement appropriateness and intervention implementation. However, several studies showed limitations in participant representativeness, control of confounding variables, and the reporting of randomization or assessor blinding. These issues do not invalidate the overall findings, but they do indicate that the pooled estimates, especially those accompanied by substantial heterogeneity, should be interpreted with appropriate caution. In particular, the variability in methodological rigor across studies may have contributed to the instability observed in some subgroup and sensitivity analyses.

### 4.2 Surprising findings

Several findings of the present study were more complex than initially anticipated. First, although the pooled effect for IO was numerically larger than that for SO, the moderator test directly comparing the two domains did not reach statistical significance. This indicates that the numerical contrast between the two pooled effects should not be interpreted in an overly literal manner. One plausible explanation is that the IO dataset was characterized by substantially greater heterogeneity and more pronounced small-study effects, thereby increasing the uncertainty surrounding the apparent advantage of IO. From a practical perspective, this suggests that GAI may indeed hold particular promise for enhancing cognitive and academic performance; however, the current evidence remains too variable to support a firm conclusion that intellectual benefits are necessarily stronger than social–emotional benefits under all conditions.

The extremely high heterogeneity observed for intellectual outcomes likely reflects the combined influence of both methodological and conceptual variation across the included studies. From a methodological perspective, the evidence base brought together randomized and quasi-experimental designs, varied substantially in sample size, intervention duration, comparator conditions, outcome timing, and disciplinary context, and used different forms of GAI integration ranging from general-purpose chatbots to more structured instructional systems. These differences are likely to have influenced the magnitude of the observed effects. From a conceptual perspective, the IO domain included a broad set of indicators, such as academic achievement, writing performance, knowledge tests, critical thinking, problem-solving, and skill-based performance. Although these outcomes all reflect intellectual or performance-related learning, they do not represent a single uniform construct. Instead, they capture related but distinct manifestations of cognitive and academic functioning. This conceptual breadth makes some degree of between-study variability expected rather than anomalous.

Importantly, the presence of such substantial heterogeneity means that the pooled estimate for IO should not be interpreted as a precise common effect size that can be generalized uniformly across all higher education contexts. Rather, it should be understood as an average directional effect across highly diverse instructional conditions and outcome measures. In practical terms, the significant pooled effect suggests that GAI is likely to benefit intellectual outcomes overall, but the exact magnitude of that benefit remains uncertain and may vary considerably depending on study design, pedagogical implementation, disciplinary setting, and the specific intellectual construct being assessed.

Second, with regard to IO, one particularly noteworthy subgroup pattern was associated with intervention duration. Although the overall moderator test did not reach the conventional threshold for statistical significance, the regression coefficient for interventions lasting more than 8 weeks was significantly larger than that for interventions lasting no more than 4 weeks. This pattern may suggest that the cognitive benefits of GAI require time to accumulate. Through repeated exposure, students may gradually learn how to formulate effective prompts, evaluate the quality of responses, incorporate feedback into the revision process, and shift from passive acceptance to more strategic use of the tool. Even so, this finding should be interpreted with caution. Subgroup estimates for the longest intervention-duration category continued to display considerable heterogeneity and statistical instability, indicating that duration alone cannot adequately explain differences in outcomes. Accordingly, the present findings do not support a simplistic “longer is always better” interpretation but rather suggest that sustained use becomes beneficial only when it is accompanied by appropriate instructional structure and effective learner regulation.

Third, a result from social–emotional analysis may seem a little counterintuitive. People may reasonably expect that more personalized tools or longer exposure will bring a stronger improvement in motivation, self-efficacy, or engagement. However, neither functional type nor intervention duration exerted a significant moderator effect on social–emotional effects. This result shows that students’ emotional and motivational responses to GAI may be affected by factors that are not directly captured in the current dataset, such as classroom climate, teacher modeling, assessment pressure, novelty effects, and students’ beliefs in authenticity and academic integrity. In other words, it may be particularly sensitive to how students understand the pedagogical meaning of artificial intelligence use, not just depending on the length of time or frequency of their use of the tool.

### 4.3 Relationship of relevant theoretical models

The results of this study can be fruitfully explained through social cognitive perspective. Social cognitive theory emphasizes that learning is shaped by the interaction between personal, behavioral, and environmental factors, and self-regulation plays a central role in goal-directed performance (Bandura, 1991). GAI can be understood as an external cognitive partner that supports self-monitoring, strategy adjustment, error detection, and the completion of complex tasks. From this perspective, its positive effect on IO is theoretically reasonable. Students who use GAI to clarify task demands, generate examples, modify drafts, or check understanding may experience more efficient self-regulation and stronger performance. The present findings are consistent with this interpretation because the strongest overall gains were observed in the domain most closely tied to strategic cognitive activity. At the same time, the high heterogeneity observed in the IO analyses also fits social cognitive theory since the effectiveness of self-regulatory support depends on learners’ prior knowledge, beliefs, goals, and capacity to evaluate the feedback they receive (Ryan and Deci, 2020).

Self-determination theory provides a second useful framework for understanding the present results (Conesa et al., 2022). According to this perspective, high-quality motivation depends on the satisfaction of the basic psychological needs for autonomy, competence, and relatedness. GAI has the potential to support autonomy by enabling flexible pacing and individualized exploration, and it may support competence by offering immediate explanatory feedback and low-stakes opportunities for practice. These characteristics help explain why SO has a milder positive effect. However, self-determination theory also shows that if students experience this technology as controlling, unreliable, or normatively ambiguous, it may also weaken motivation. A tool that can both help and cause dependency, surveillance, or academic dishonesty concerns may not really support need satisfaction. This theoretical framework helps to explain why the SOs in this study are less than IO, and why these effects do not systematically vary across the main moderator category. This means that social–emotional benefits may depend less on the existence of GAI itself but more on whether the instructional context supports students’ agency, competence, and meaningful participation.

The third explanatory perspective comes from feedback theory and generative learning theory (Hattie and Timperley, 2007). Effective feedback can promote learning when it clarifies goals, points out current progress, and guides follow-up improvement. GAI is particularly suitable for performing these functions at a higher speed and with obvious personalization (Fiorella and Mayer, 2016). At the same time, generative learning theory believes that when students take the initiative to organize, integrate, explain, and elaborate information, deep learning can occur. These two theoretical traditions together help to explain why GAI may improve IO when used as a tool for revision, explanation, and iterative dialogue. It can provide frequent informational feedback, but its educational value depends on whether students actively participate in and process this feedback, rather than just copying outputs. This also provides a useful perspective for explaining the heterogeneity observed in this study. When GAI is used to stimulate the active reconstruction of knowledge, its effect may be positive and significant, and when it is mainly used as a shortcut to replace cognitive effort rather than a tool to support cognitive effort, its impact may be limited and may even be counterproductive. Therefore, the broader theoretical picture revealed by the results of this study is that GAI should not be understood as an autonomous instructional solution but should be regarded as a pedagogical amplifier. The effect depends on the quality of human design, the level of learner engagement, and whether it provides opportunities for reflective processing.

### 4.4 Strengths and limitations

This research has several advantages. First of all, this study focuses on higher education, which improves conceptual consistency and avoids the developmental heterogeneity that may occur after combining school and university situations. Second, this study distinguishes between IO and SO so that it can more carefully assess which aspects GAI is most likely to benefit. Third, the comprehensive analysis of this study is based on experimental and quasi-experimental evidence, rather than relying mainly on the investigation of perceptions or intentions. This enhances the relevance of the research results to the actual educational effect. Fourth, this study combines overall meta-analysis, publication bias analysis, sensitivity analysis, and moderator analysis so that the current evidence base can be explained more distinctly.

At the same time, it should also be recognized that there are some limitations of this study. A major limitation of the present study is the substantial heterogeneity observed across several analyses, particularly for intellectual outcomes. This level of heterogeneity suggests that the included studies differed not only in methodological features, such as design, intervention format, duration, and comparator conditions but also in the specific constructs used to represent intellectual performance. Consequently, confidence in the pooled estimate for IO should be moderated. Although the overall direction of the effect was positive, the exact size of the pooled effect is less certain and should be interpreted as a broad summary tendency rather than a highly stable common parameter. This shows that important variable sources have not yet been measured or have not been fully captured by the moderator variables available in the current dataset. This high heterogeneity reduces confidence in the pooled IO estimates and suggests that the corresponding effect sizes should be interpreted with caution. The second limitation involves the evidence of publication bias and small-study effects in IO literature, which means that the corresponding combined effects may be overestimated. Third, the number of studies included in some subgroup analyses is still limited, especially in terms of SO, which reduces statistical power and makes it necessary for moderators to do category consolidation. Fourth, although this study introduces theoretically meaningful moderator coding, the existing primary studies often lack instructional design, teacher involvement, and the degree of learner autonomy. As a result, several potentially important moderators could not be examined directly in the present meta-analysis. Finally, the field itself is still developing rapidly, and many of the included studies focus on the early implementation of tools closely related to ChatGPT. Therefore, the results of this study should be regarded as a reference but still a temporary discovery, especially in terms of moderator patterns and long-term educational impact, and they should therefore be interpreted with particular caution. In addition, although the review followed a prospectively registered protocol, minor reporting refinements were introduced in the final manuscript to improve conceptual clarity and transparency. These changes did not affect the main analytical framework or the overall conclusions of the study.

## 5 Conclusion

This system review and meta-analysis synthesizes the evidence of 33 experimental and quasi-experimental studies to examine GAI for higher education on the influence of IO and SO. The research results show that GAI has a significant positive effect on both fields. In general, the effect of GAI on IO is numerically greater than its effect on social SO, but the difference between the two areas does not reach statistical significance. These results show that GAI has important potential in supporting the learning of higher education students, especially in academic and cognitive performance.

At the same time, this study also demonstrates that the current evidence base remains highly heterogeneous, particularly with respect to IO, and that publication bias and small-study effects may have influenced the magnitude of some pooled estimates. Moderator analyses further indicate that, at the omnibus level, functional type, intervention duration, and knowledge domain do not consistently account for between-study variation although several subgroup trends remain noteworthy. This pattern suggests that the educational effects of GAI are unlikely to depend solely on the technology itself. Rather, these effects appear to be shaped more substantially by the quality of instructional design, the nature of learning tasks, and the ways in which artificial intelligence tools are pedagogically integrated into higher education settings.

Overall, the findings of this study support a more balanced interpretation. GAI should neither be viewed as a universal solution nor dismissed as an inherently problematic disruptive force. Instead, it should be understood as a potentially valuable educational resource whose benefits depend on thoughtful implementation, critical use, and alignment with pedagogical goals. Future research should place greater emphasis on more rigorous experimental designs, longer intervention durations, clearer theoretical grounding, and more detailed reporting of instructional conditions, so that the mechanisms and boundary conditions of GAI in higher education can be understood with greater precision.

## Data Availability

The original contributions presented in the study are included in the article/Supplementary material, further inquiries can be directed to the corresponding author/s.
