# Supplementary material for: Generative AI in higher education: a meta-analysis of intellectual and social–emotional outcomes
Source: Front Psychol. 2026 Jun 8;17:1848745. doi: 10.3389/fpsyg.2026.1848745 (PMC13284697; doi:10.3389/fpsyg.2026.1848745)
Supplement: Supplementary file 1 [file Supplementary_file_1.docx]

Generative AI in Higher Education: A Meta-Analysis of Intellectual and Social-Emotional Outcomes

Chunai Liu^1*^, Lidong Xie^1^, and Gang Xu^2*^

**Supplemental Table 1**. Conceptualization of key outcome domains and representative measurement indicators in prior GAI research in higher education

| Outcome domain | Conceptual definition | Representative constructs | Typical measurement indicators |
| --- | --- | --- | --- |
| Intellectual outcomes | Performance-oriented cognitive and academic consequences of learning | Academic achievement, knowledge acquisition, writing performance, feedback quality, problem-solving, critical thinking, discipline-specific task performance | Test scores, course assessments, rubric-based writing scores, task performance ratings, achievement measures |
| Social-emotional outcomes | Affective, motivational, and self-perceptual consequences of learning | Motivation, engagement, attitudes, self-efficacy, enjoyment, anxiety, willingness to communicate | Self-report questionnaires, Likert-scale surveys, validated motivational or affective scales |

**Supplemental Table 2**. Summary of features of included intervention

| **Study** | **country** | **paper type** | **Experimental Group** | **Control Group** | **Functional types of GAI** | **Outcome** | **Outcome Measures** | **Intervention duration** | **Knowledge domains** |
| --- | --- | --- | --- | --- | --- | --- | --- | --- | --- |
| Yin et al, 2021 | China | Quasi-experimental | 51 | 48 | Personalized learning support | SO | Motivation questionnaire | ≤4 weeks | Information and Communication Technologies |
| V´azquez-Cano et al, 2021 | Spain | Quasi-experimental | 52 | 51 | Personalized tutoring systems | IO | Final test | ﹥8 weeks | Arts and Humanities |
| Mirzababaei & Pammer-Schindler, 2022 | Austria | Quasi-experimental | 42 | 53 | Personalized tutoring systems | IO | Final task | ≤4 weeks | Information and Communication Technologies |
| Kim & Lee, 2022 | South Korea | Quasi-experimental | 160 | 160 | Assessment | IO | Questionnaires | ≤4 weeks | Arts and Humanities |
| Kuo & Chen, 2022 | China | Quasi-experimental | 30 | 30 | Assessment | SO | Questionnaires | ≤4 weeks | Information and Communication Technologies |
| Lee et al, 2022 | China | Quasi-experimental | 18 | 20 | Personalized learning support | SO | Measurement form | ≤4 weeks | Health amd Welfare |
| Essel et al, 2022 | Ghana | Pretest–posttest | 34 | 34 | Assessment | IO | Achievement test | ﹥8 weeks | Information and Communication Technologies |
| Han et al, 2022 | Korea | Quasi-experimental | 30 | 31 | Assessment | IO | Questionnaires | ≤4 weeks | Health amd Welfare |
| Escalante et al, 2023 | USA | Quasi-experimental | 23 | 25 | Assessment | IO | Writing assignment | ﹥4-8 weeks | Arts and Humanities |
| Yilmaz & Yilmaz, 2023 | Turkey | Pretest-posttest | 21 | 24 | Assessment | IO | Homework | ﹥4-8 weeks | Information and Communication Technologies |
| Al Kahf et al, 2023 | France | Randomized controlled trial | 213 | 171 | Personalized learning support | IO | Questionnaires | ﹥4-8 weeks | Arts and Humanities |
| li, 2023 | China | Quasi-experimental | 42 | 39 | Personalized tutoring systems | IO | Questionnaires | ≤4 weeks | Education |
| Liu et al, 2023 | China | Quasi-experimental | 31 | 37 | Personalized learning support | SO | Questionnaires | ≤4 weeks | Education |
| Zhang & Cheng, 2023 | China | Quasi-experimental | 15 | 15 | Personalized tutoring systems | SO | Questionnaires | ﹥4-8 weeks | Education |
| Hakiki et al, 2023 | Indonesia | Quasi-experimental | 31 | 31 | Personalized tutoring systems | IO | Questionnaires | ≤4 weeks | Education |
| Silitonga et al, 2023 | Indonesia | Quasi-experimental | 36 | 37 | Personalized tutoring systems | SO | Questionnaires | ﹥4-8 weeks | Arts and Humanities |
| Qureshi, 2023 | Saudi Arabia | Quasi-experimental | 12 | 12 | Personalized learning support | IO | Questionnaires | ≤4 weeks | Information and Communication Technologies |
| Sadek & Mohamed, 2023 | Egypt | Pretest-posttest | 22 | 47 | Personalized learning support | IO | Online surveys | ﹥8 weeks | Engineering, manufacturing and construction |
| Pellas, 2023 | Greece | Pretest-posttest | 32 | 32 | Personalized learning support | SO | Intelligence questionnaire | ﹥4-8 weeks | Education |
| Song & Song, 2023 | China | Pretest-posttest | 25 | 25 | Personalized learning support | SO | Questionnaires | ﹥8 weeks | Arts and Humanities |
| Li et al, 2024 | China | Pretest-posttest | 30 | 31 | Personalized learning support | IO | Questionnaires | ﹥4-8 weeks | Arts and Humanities |
| Saritepeci & Durak, 2024 | Turkey | Pretest-posttest | 87 | 99 | Personalized learning support | IO | Questionnaires | ﹥4-8 weeks | Education |
| Wang & Xue, 2024 | China | Pretest-posttest | 57 | 56 | Personalized learning support | SO | Questionnaires | ≤4 weeks | Arts and Humanities |
| Yin et al, 2024 | China | Pretest-posttest | 88 | 85 | Assessment | SO | Questionnaires | ≤4 weeks | Science |
| Bhatia et al, 2024 | India | Pretest-posttest | 82 | 82 | Personalized tutoring systems | IO | Questionnaires | ≤4 weeks | Health amd Welfare |
| Chen & Hou, 2024 | China | Pretest-posttest | 32 | 29 | Personalized learning support | SO | Questionnaires | ﹥4-8 weeks | Education |
| Essel et al, 2024 | Ghana | Pretest-posttest | 60 | 65 | Personalized learning support | IO | Questionnaires | ≤4 weeks | Education |
| Guo et al, 2024 | China | Pretest-posttest | 64 | 60 | Assessment | IO | Questionnaires | ﹥8 weeks | Education |
| Boudouaia et al, 2024 | China | Pretest-posttest | 37 | 39 | Personalized tutoring systems | IO | Questionnaires | ﹥8 weeks | Arts and Humanities |
| Fathi et al, 2024 | Iran | Pretest-posttest | 33 | 32 | Personalized tutoring systems | IO | Questionnaires | ﹥8 weeks | Arts and Humanities |
| Zhang et al, 2024 | China | Pretest-posttest | 65 | 66 | Personalized learning support | IO | Questionnaires | ﹥8 weeks | Arts and Humanities |
| Shi et al, 2024 | China | Pretest-posttest | 64 | 64 | Assessment | IO | Questionnaires | ≤4 weeks | Business, administration and law |
| Urban et al, 2024 | Czech | Pretest-posttest | 77 | 68 | Assessment | SO | Questionnaires | ≤4 weeks | NA |

**Supplementary Table 3.** Retained effect sizes and decision rules used to address non-independence in the quantitative synthesis.

| Study | Reported eligible outcomes | Domain(s) reported | Outcome retained for meta-analysis | Time point retained | Non-independence issue | Decision rule applied |
| --- | --- | --- | --- | --- | --- | --- |
| Yin et al., 2021 | Single eligible SO outcome reported in the article and extracted in Supplementary Table 2 | SO only | Motivation questionnaire | End-of-intervention post-test | No major non-independence issue | Single eligible SO effect retained |
| Vázquez-Cano et al., 2021 | Single eligible IO outcome reported in the article and extracted in Supplementary Table 2 | IO only | Final test | End-of-intervention post-test | No major non-independence issue | Single eligible IO effect retained |
| Mirzababaei and Pammer-Schindler, 2022 | Single eligible IO outcome reported in the article and extracted in Supplementary Table 2 | IO only | Final task | End-of-intervention task assessment | No major non-independence issue | Single eligible IO effect retained |
| Kim and Lee, 2022 | Single eligible IO outcome reported in the article and extracted in Supplementary Table 2 | IO only | Questionnaire-based IO outcome | Post-intervention assessment | No major non-independence issue | Single eligible IO effect retained |
| Kuo and Chen, 2022 | Single eligible SO outcome reported in the article and extracted in Supplementary Table 2 | SO only | Questionnaire-based SO outcome | Post-test | No major non-independence issue | Single eligible SO effect retained |
| Lee et al., 2022 | Single eligible SO outcome reported in the article and extracted in Supplementary Table 2 | SO only | Measurement-form SO outcome | Post-test | No major non-independence issue | Single eligible SO effect retained |
| Essel et al., 2022 | Single eligible IO outcome reported in the article and extracted in Supplementary Table 2 | IO only | Achievement test | Post-test | No major non-independence issue | Single eligible IO effect retained |
| Han et al., 2022 | Single eligible IO outcome reported in the article and extracted in Supplementary Table 2 | IO only | Questionnaire-based IO outcome | Post-test | No major non-independence issue | Single eligible IO effect retained |
| Escalante et al., 2023 | Study 1 writing performance outcome and Study 2 weekly preference survey | IO and non-eligible ancillary preference component | Writing assignment from Study 1 | Final post-intervention post-test | Multiple reported components within the same article | Only the outcome aligned with the review framework and eligible independent comparison was retained |
| Yilmaz and Yilmaz, 2023 | Single eligible IO outcome reported in the article and extracted in Supplementary Table 2 | IO only | Homework outcome | Post-test | No major non-independence issue | Single eligible IO effect retained |
| Al Kahf et al., 2023 | Single eligible IO outcome reported in the article and extracted in Supplementary Table 2 | IO only | Questionnaire-based IO outcome | Post-test | No major non-independence issue | Single eligible IO effect retained |
| Li, 2023 | Single eligible IO outcome reported in the article and extracted in Supplementary Table 2 | IO only | Questionnaire-based IO outcome | Post-test | No major non-independence issue | Single eligible IO effect retained |
| Liu et al., 2023 | Single eligible SO outcome reported in the article and extracted in Supplementary Table 2 | SO only | Questionnaire-based SO outcome | Post-test | No major non-independence issue | Single eligible SO effect retained |
| Zhang and Cheng, 2023 | Single eligible SO outcome reported in the article and extracted in Supplementary Table 2 | SO only | Questionnaire-based SO outcome | Post-test | No major non-independence issue | Single eligible SO effect retained |
| Hakiki et al., 2023 | Single eligible IO outcome reported in the article and extracted in Supplementary Table 2 | IO only | Questionnaire-based IO outcome | Post-test | No major non-independence issue | Single eligible IO effect retained |
| Silitonga et al., 2023 | Single eligible SO outcome reported in the article and extracted in Supplementary Table 2 | SO only | Questionnaire-based SO outcome | Post-test | No major non-independence issue | Single eligible SO effect retained |
| Qureshi, 2023 | Single eligible IO outcome reported in the article and extracted in Supplementary Table 2 | IO only | Questionnaire-based IO outcome | Post-test | No major non-independence issue | Single eligible IO effect retained |
| Sadek and Mohamed, 2023 | Single eligible IO outcome reported in the article and extracted in Supplementary Table 2 | IO only | Online survey-based IO outcome | End-of-intervention post-test | No major non-independence issue | Single eligible IO effect retained |
| Pellas, 2023 | Single eligible SO outcome reported in the article and extracted in Supplementary Table 2 | SO only | Intelligence questionnaire | Post-test | No major non-independence issue | Single eligible SO effect retained |
| Song and Song, 2023 | Global writing performance, writing content, writing organization, writing language use, and writing motivation | IO and SO | Writing motivation questionnaire | Final post-test | Both IO and SO reported, with multiple writing-related performance indicators | One SO effect was retained for the SO dataset; writing subdimensions were not entered separately |
| Li et al., 2024 | Single eligible IO outcome reported in the article and extracted in Supplementary Table 2 | IO only | Questionnaire-based IO outcome | Post-test | No major non-independence issue | Single eligible IO effect retained |
| Saritepeci and Durak, 2024 | Single eligible IO outcome reported in the article and extracted in Supplementary Table 2 | IO only | Questionnaire-based IO outcome | Post-test | No major non-independence issue | Single eligible IO effect retained |
| Wang and Xue, 2024 | Single eligible SO outcome reported in the article and extracted in Supplementary Table 2 | SO only | Questionnaire-based SO outcome | Post-test | No major non-independence issue | Single eligible SO effect retained |
| Yin et al., 2024 | Single eligible SO outcome reported in the article and extracted in Supplementary Table 2 | SO only | Questionnaire-based SO outcome | Post-test | No major non-independence issue | Single eligible SO effect retained |
| Bhatia et al., 2024 | Single eligible IO outcome reported in the article and extracted in Supplementary Table 2 | IO only | Questionnaire-based IO outcome | Post-test | No major non-independence issue | Single eligible IO effect retained |
| Chen and Hou, 2024 | Ethics knowledge pretest-posttest outcome together with flow, motivation, anxiety, and related questionnaire-based measures | IO and SO | Questionnaire-based SO outcome | Final post-intervention assessment | Both IO and SO reported from the same sample | One SO effect was retained for the SO dataset; knowledge test outcomes were not entered simultaneously |
| Essel et al., 2024 | Single eligible IO outcome reported in the article and extracted in Supplementary Table 2 | IO only | Questionnaire-based IO outcome | Post-test | No major non-independence issue | Single eligible IO effect retained |
| Guo et al., 2024 | Single eligible IO outcome reported in the article and extracted in Supplementary Table 2 | IO only | Questionnaire-based IO outcome | Post-test | No major non-independence issue | Single eligible IO effect retained |
| Boudouaia et al., 2024 | Single eligible IO outcome reported in the article and extracted in Supplementary Table 2 | IO only | Questionnaire-based IO outcome | Post-test | No major non-independence issue | Single eligible IO effect retained |
| Fathi et al., 2024 | Single eligible IO outcome reported in the article and extracted in Supplementary Table 2 | IO only | Questionnaire-based IO outcome | Post-test | No major non-independence issue | Single eligible IO effect retained |
| Zhang et al., 2024 | Single eligible IO outcome reported in the article and extracted in Supplementary Table 2 | IO only | Questionnaire-based IO outcome | Post-test | No major non-independence issue | Single eligible IO effect retained |
| Shi et al., 2024 | Learning effectiveness, cognitive dimension, skill dimension, affective dimension, flow experience, and sentiment evaluations | IO and SO | Overall learning effectiveness questionnaire | Final post-intervention assessment | Multiple IO subdimensions and additional SO indicators reported from the same sample | Overall IO measure was retained over subdimensions; one independent effect was entered |
| Urban et al., 2024 | Quality, elaboration, originality, self-efficacy, self-evaluation accuracy, task interest, task difficulty, and mental effort | IO and SO | Questionnaire-based SO outcome | End-of-task post-test | Both IO and SO reported, with multiple related indicators from the same sample | One SO effect was retained for the SO dataset; performance indicators and other questionnaire outcomes were not entered simultaneously |

**Note.** For studies reporting a single eligible outcome aligned with the review framework, that single effect size was retained. For studies reporting multiple eligible outcomes, only one independent effect size per study was entered into the quantitative synthesis according to prespecified rules, prioritizing conceptual centrality, comparability with the broader evidence base, overall scores over subscales where appropriate, and the final post-intervention endpoint. IO = intellectual outcomes. SO = social-emotional outcomes.

**Supplemental Table 4**. Meta analytic summary for IO and SO.

| **Outcome domain** | **Number of studies** | **Model** | **Pooled Hedges’s g** | **95% confidence interval** | **z value** | **p value** | **tau squared** | **I squared (%)** | **H squared** | **Q value** | **Heterogeneity p value** |
| --- | --- | --- | --- | --- | --- | --- | --- | --- | --- | --- | --- |
| IO | 21 | Random effects model using REML | 1.096 | 0.087 to 2.104 | 2.13 | 0.0332 | 5.4588 | 99.27 | 136.51 | 454.41 | <0.001 |
| SO | 12 | Random effects model using REML | 0.301 | 0.048 to 0.553 | 2.34 | 0.0195 | 0.1395 | 73.2 | 3.73 | 42.35 | <0.001 |

**Supplemental Table 5**. Publication bias tests for IO and SO.

| **Outcome domain** | **Funnel plot assessment** | **Egger beta1** | **Egger standard error** | **Egger z value** | **Egger p value** | **Begg Kendall’s score** | **Begg standard error** | **Begg z value** | **Begg p value** | **Interpretation** |
| --- | --- | --- | --- | --- | --- | --- | --- | --- | --- | --- |
| IO | Visual asymmetry observed | 12.6 | 1.53 | 8.23 | <0.001 | 110 | 33.116 | 3.29 | 0.001 | Evidence of possible publication bias or small study effects |
| SO | No marked asymmetry observed | 1.92 | 2.26 | 0.85 | 0.3967 | 12 | 14.583 | 0.75 | 0.4507 | No substantial evidence of publication bias |

**Supplemental Table 6**. Leave one out sensitivity analysis for IO.

| **Omitted study** | **Recalculated Hedges’s g** | **95% confidence interval** | **p value** |
| --- | --- | --- | --- |
| Vázquez Cano et al., 2021 | 1.135 | 0.071 to 2.199 | 0.037 |
| Mirzababaei and Pammer Schindler, 2022 | 1.127 | 0.062 to 2.193 | 0.038 |
| Kim and Lee, 2022 | 1.142 | 0.079 to 2.205 | 0.035 |
| Essel et al., 2022 | 0.922 | -0.067 to 1.910 | 0.068 |
| Han et al., 2022 | 1.123 | 0.058 to 2.189 | 0.039 |
| Escalante et al., 2023 | 1.161 | 0.104 to 2.219 | 0.031 |
| Yilmaz and Yilmaz, 2023 | 1.106 | 0.039 to 2.172 | 0.042 |
| Al Kahf et al., 2023 | 1.14 | 0.077 to 2.204 | 0.036 |
| Li, 2023 | 1.13 | 0.065 to 2.195 | 0.038 |
| Hakiki et al., 2023 | 1.084 | 0.018 to 2.149 | 0.046 |
| Qureshi, 2023 | 1.108 | 0.043 to 2.173 | 0.042 |
| Sadek and Mohamed, 2023 | 1.149 | 0.088 to 2.210 | 0.034 |
| Li et al., 2024 | 1.112 | 0.045 to 2.178 | 0.041 |
| Saritepeci and Durak, 2024 | 1.15 | 0.089 to 2.212 | 0.034 |
| Bhatia et al., 2024 | 1.242 | 0.229 to 2.255 | 0.016 |
| Essel et al., 2024 | 1.153 | 0.093 to 2.214 | 0.033 |
| Guo et al., 2024 | 1.06 | -0.002 to 2.123 | 0.05 |
| Boudouaia et al., 2024 | 0.625 | 0.140 to 1.110 | 0.011 |
| Fathi et al., 2024 | 1.102 | 0.036 to 2.168 | 0.043 |
| Zhang et al., 2024 | 1.135 | 0.071 to 2.199 | 0.037 |
| Shi et al., 2024 | 1.14 | 0.077 to 2.204 | 0.036 |
| Original pooled estimate | 1.096 | 0.087 to 2.104 | 0.033 |

**Supplemental Table 7**. Leave one out sensitivity analysis for SO.

| **Omitted study** | **Pooled Hedges’s g** | **95% CI** | **p value** |
| --- | --- | --- | --- |
| Yin et al., 2021 | 0.328 | 0.055 to 0.601 | 0.018 |
| Kuo and Chen, 2022 | 0.269 | 0.003 to 0.535 | 0.047 |
| Lee et al., 2022 | 0.268 | 0.006 to 0.529 | 0.045 |
| Liu et al., 2023 | 0.313 | 0.038 to 0.589 | 0.026 |
| Zhang and Cheng, 2023 | 0.321 | 0.054 to 0.588 | 0.019 |
| Silitonga et al., 2023 | 0.222 | -0.004 to 0.448 | 0.054 |
| Pellas, 2023 | 0.309 | 0.033 to 0.584 | 0.028 |
| Song and Song | 0.283 | 0.012 to 0.554 | 0.04 |
| Wang and Xue, 2024 | 0.28 | 0.005 to 0.554 | 0.046 |
| Yin et al., 2024 | 0.369 | 0.130 to 0.608 | 0.002 |
| Chen and Hou, 2024 | 0.358 | 0.112 to 0.604 | 0.004 |
| Urban et al., 2024 | 0.288 | 0.010 to 0.567 | 0.042 |
| Original pooled estimate | 0.301 | 0.048 to 0.553 | 0.02 |

**Supplemental Table 8**. Moderator analysis of functional types of GAI for IO.

| **Functional type of GAI** | **Number of studies** | **Pooled Hedges’s g** | **95% confidence interval** | **z value** | **p value** | **I squared (%)** | **Q value** | **Heterogeneity p value** |
| --- | --- | --- | --- | --- | --- | --- | --- | --- |
| Assessment | 7 | 1.168 | 0.019 to 2.316 | 1.99 | 0.0463 | 98.03 | 130.38 | <0.001 |
| Personalized learning support | 7 | 0.291 | 0.099 to 0.483 | 2.96 | 0.003 | 47.16 | 11.99 | 0.0622 |
| Personalized tutoring systems | 7 | 1.858 | -1.242 to 4.958 | 1.17 | 0.2401 | 99.67 | 294.56 | <0.001 |

**Note:** The omnibus test from the random effects meta regression was not statistically significant, with Wald chi square = 1.10 and p = 0.5759. Assessment served as the reference category. Compared with assessment, the coefficient for personalized learning support was -0.805, with a standard error of 1.303, a 95% confidence interval from -3.358 to 1.748, and p = 0.537. The coefficient for personalized tutoring systems was 0.561, with a standard error of 1.308, a 95% confidence interval from -2.004 to 3.125, and p = 0.668. Residual tau squared was 5.871, residual I squared was 99.27%, and R squared was 0.00%.

**Supplemental Table 9.** Moderator analysis of intervention duration for IO.

| **Intervention duration** | **Number of studies** | **Pooled Hedges’s g** | **95% confidence interval** | **z value** | **p value** | **I squared (%)** | **Q value** | **Heterogeneity p value** |
| --- | --- | --- | --- | --- | --- | --- | --- | --- |
| Less than or equal to 4 weeks | 9 | 0.281 | negative 0.314 to 0.876 | 0.93 | 0.3546 | 95.22 | 150.55 | <0.001 |
| Greater than 4 to 8 weeks | 5 | 0.378 | negative 0.007 to 0.764 | 1.93 | 0.0542 | 80.2 | 13.99 | 0.0073 |
| More than 8 weeks | 7 | 2.755 | negative 0.181 to 5.692 | 1.84 | 0.0659 | 99.58 | 229.56 | <0.001 |

**Note:** The omnibus test from the random effects meta regression did not reach statistical significance, with Wald chi square = 4.96 and p = 0.0836. The reference category was less than or equal to 4 weeks. Relative to this category, the coefficient for greater than 4 to 8 weeks was 0.113, with a standard error of 1.227, a 95% confidence interval from negative 2.293 to 2.518, and p = 0.927. The coefficient for more than 8 weeks was 2.327, with a standard error of 1.117, a 95% confidence interval from 0.138 to 4.516, and p = 0.037. Residual tau squared was 4.785, residual I squared was 99.09%, and R squared was 12.34%.

**Supplemental Table 10.** Moderator analysis of knowledge domain for IO.

| **Knowledge domain** | **Number of studies** | **Pooled Hedges’s g** | **95% confidence interval** | **z value** | **p value** | **I squared (%)** | **Q value** | **Heterogeneity p value** |
| --- | --- | --- | --- | --- | --- | --- | --- | --- |
| Applied and Technical Disciplines | 8 | 0.741 | negative 0.460 to 1.942 | 1.21 | 0.2265 | 97.91 | 225.94 | <0.001 |
| Arts and Humanities | 4 | 3.372 | negative 1.781 to 8.524 | 1.28 | 0.1996 | 99.72 | 130.28 | <0.001 |
| Education and Humanities | 9 | 0.505 | 0.071 to 0.940 | 2.28 | 0.0227 | 89.38 | 75.33 | <0.001 |

**Note:** The omnibus test from the random effects meta regression was not statistically significant, with Wald chi square = 3.88 and p = 0.1439. Applied and Technical Disciplines served as the reference category. Relative to this category, the coefficient for Arts and Humanities was 2.386, with a standard error of 1.412, a 95% confidence interval from negative 0.381 to 5.153, and p = 0.091. The coefficient for Education and Humanities was negative 0.237, with a standard error of 1.107, a 95% confidence interval from negative 2.406 to 1.932, and p = 0.830. Residual tau squared was 5.122, residual I squared was 99.20%, and R squared was 6.18%.

**Supplemental Table 11.** Moderator analysis of functional types of GAI for SO.

| **Functional type of GAI** | **Number of studies** | **Pooled Hedges’s g** | **95% confidence interval** | **z value** | **p value** | **I squared (%)** | **Q value** | **Heterogeneity p value** |
| --- | --- | --- | --- | --- | --- | --- | --- | --- |
| Personalized learning support | 7 | 0.245 | negative 0.017 to 0.507 | 1.83 | 0.0673 | 52.83 | 12.76 | 0.0471 |
| Structured systems | 5 | 0.377 | negative 0.123 to 0.877 | 1.48 | 0.1397 | 85.2 | 29.58 | <0.001 |

**Note:** The omnibus test from the random effects meta regression was not statistically significant, with Wald chi square = 0.19 and p = 0.6601. Personalized learning support served as the reference category. Relative to this category, the coefficient for Structured systems was 0.119, with a standard error of 0.271, a 95% confidence interval from negative 0.412 to 0.650, and p = 0.660. Residual tau squared was 0.1557, residual I squared was 74.71%, and R squared was 0.00%.

**Supplemental Table 12.** Moderator analysis of intervention duration for SO.

| **Intervention duration** | **Number of studies** | **Pooled Hedges’s g** | **95% confidence interval** | **z value** | **p value** | **I squared (%)** | **Q value** | **Heterogeneity p value** |
| --- | --- | --- | --- | --- | --- | --- | --- | --- |
| Less than or equal to 4 weeks | 7 | 0.286 | 0.000 to 0.572 | 1.96 | 0.0496 | 70.73 | 22.25 | 0.0011 |
| More than 4 weeks | 5 | 0.307 | negative 0.206 to 0.821 | 1.17 | 0.2404 | 78.09 | 19.45 | 0.0006 |

**Note:** The omnibus test from the random effects meta regression was not statistically significant, with Wald chi square = 0.00 and p = 0.9572. The reference category was less than or equal to 4 weeks. Relative to this category, the coefficient for more than 4 weeks was 0.015, with a standard error of 0.277, a 95% confidence interval from negative 0.527 to 0.557, and p = 0.957. Residual tau squared was 0.158, residual I squared was 75.64%, and R squared was 0.00%.

**Supplemental Table 13.** Pooled effects of GAI across outcome domains.

| **Outcome domain** | **Number of studies** | **Hedges’s g** | **95% CI** | **z** | **p value** | **I² (%)** | **Q** | **Heterogeneity p value** |
| --- | --- | --- | --- | --- | --- | --- | --- | --- |
| IO | 21 | 1.096 | 0.087 to 2.104 | 2.13 | 0.0332 | 99.27 | 454.41 | <0.001 |
| SO | 12 | 0.301 | 0.048 to 0.553 | 2.34 | 0.0195 | 73.2 | 42.35 | <0.001 |

**Supplemental Table 14.** Meta regression examining outcome domain as a moderator.

| **Moderator comparison** | **Coefficient** | **Standard error** | **z** | **p value** | **95% CI** | **Wald chi square** | **Omnibus p value** | **Residual tau²** | **Residual I² (%)** | **R² (%)** |
| --- | --- | --- | --- | --- | --- | --- | --- | --- | --- | --- |
| SO relative to IO | -0.739 | 0.645 | -1.15 | 0.252 | -2.004 to 0.526 | 1.31 | 0.2522 | 3.105 | 98.62 | 0 |

**Supplementary Table 15.** Methodological quality appraisal of the included studies using the Mixed Methods Appraisal Tool, MMAT, 2018

| Study | MMAT category | C1 Representativeness | C2  Appropriate measurements | C3  Complete outcome data | C4  Confounders accounted for | C5  Intervention administered as intended |
| --- | --- | --- | --- | --- | --- | --- |
| Yin et al., 2021 | Non-randomized | Yes | Yes | Yes | Yes | Yes |
| Vázquez-Cano et al., 2021 | Non-randomized | Yes | Yes | Yes | Yes | Yes |
| Mirzabaeei and Pammer-Schindler, 2022 | Non-randomized | Can’t tell | Yes | Can’t tell | Can’t tell | Yes |
| Kim and Lee, 2022 | Non-randomized | No | Yes | Yes | Yes | Yes |
| Kuo and Chen, 2022 | Non-randomized | Can’t tell | Yes | Yes | Yes | Yes |
| Lee et al., 2022 | Non-randomized | Can’t tell | Yes | Yes | Yes | Yes |
| Essel et al., 2022 | Non-randomized | Yes | Yes | Yes | Yes | Yes |
| Han et al., 2022 | Non-randomized | Can’t tell | Yes | Yes | Yes | Yes |
| Escalante et al., 2023 | Non-randomized | No | Yes | Yes | Yes | Yes |
| Yilmaz and Yilmaz, 2023 | Non-randomized | Can’t tell | Yes | No | Can’t tell | Yes |
| Al Kahf et al., 2023 | Randomized | Can’t tell | Yes | Yes | Can’t tell | No |
| Li, 2023 | Non-randomized | Can’t tell | Yes | Yes | Yes | Yes |
| Liu et al., 2024 | Non-randomized | Can’t tell | Yes | Yes | Yes | Yes |
| Zhang & Cheng, 2023 | Non-randomized | No | Yes | Yes | No | Yes |
| Hakiki et al., 2023 | Non-randomized | Can’t tell | Yes | Yes | Can’t tell | Yes |
| Silitonga et al., 2023 | Non-randomized | Can’t tell | Yes | Yes | Yes | Yes |
| Qureshi, 2023 | Non-randomized | Can’t tell | Yes | Yes | Yes | Yes |
| Sadek and Mohamed, 2023 | Non-randomized | No | Yes | No | No | Yes |
| Pellas, 2023 | Non-randomized | No | Yes | Yes | Yes | Yes |
| Song and Song, 2023 | Non-randomized | Yes | Yes | Yes | Can’t tell | Yes |
| Li et al., 2024 | Non-randomized | Can’t tell | Yes | Yes | Yes | Yes |
| Saritepeci and Durak, 2024 | Non-randomized | Can’t tell | Yes | Yes | Can’t tell | Yes |
| Wang and Xue, 2024 | Non-randomized | Can’t tell | Yes | Yes | Can’t tell | Yes |
| Yin et al., 2024 | Non-randomized | Can’t tell | Yes | Yes | Yes | Yes |
| Bhatia et al., 2024 | Randomized | Yes | Can’t tell | No | Can’t tell | Can’t tell |
| Chen and Hou, 2024 | Non-randomized | No | Yes | Yes | Yes | Yes |
| Essel et al., 2024 | Randomized | Yes | Yes | Yes | Can’t tell | Yes |
| Guo et al., 2024 | Non-randomized | Can’t tell | Yes | Yes | Yes | Yes |
| Boudouaia et al., 2024 | Randomized | Can’t tell | Can’t tell | Yes | Can’t tell | Yes |
| Fathi et al., 2024 | Randomized | Yes | Yes | Yes | Can’t tell | Yes |
| Zhang et al, 2024 | Non-randomized | Can’t tell | Yes | No | Yes | No |
| Shi et al., 2024 | Randomized | Can’t tell | Yes | Yes | Can’t tell | Yes |
| Urban et al., 2024 | Randomized | Yes | Yes | Yes | Yes | Yes |

Note. For randomized studies, Criterion 1 indicates whether randomization was appropriately performed, Criterion 2 baseline comparability, Criterion 3 completeness of outcome data, Criterion 4 blinding of outcome assessors, and Criterion 5 adherence to the assigned intervention. For non-randomized studies, Criterion 1 indicates representativeness of the target population, Criterion 2 appropriateness of measurements, Criterion 3 completeness of outcome data, Criterion 4 whether confounders were accounted for, and Criterion 5 whether the intervention was administered as intended.
